# Mast cell–expressed Mrgprb2/MRGPRX2 mediates gout pain and inflammation via a neuroimmune axis

**DOI:** 10.1172/jci.insight.201781

**Published:** 2026-01-23

**Authors:** Lin Yang, Chengxi Liu, Jin Xiao, Yu Song, Huan Chen, Dan Li, Cong Zou, Tao Hong, Yinglan Liu, Dake Qi, Nathachit Limjunyawong, Wenjie Liu, Lintao Qu

**Affiliations:** 1 Anesthesiology and Pain Research Institute, Second Affiliated Hospital,; 2 Hengyang Medical School,; 3 Department of Anesthesiology, Second Affiliated Hospital,; 4 Department of Anesthesiology, First Affiliated Hospital, and; 5 Department of Endocrinology, Second Affiliated Hospital, University of South China, Hengyang, Hunan, China.; 6 College of Pharmacy, Rady Faculty of Health Sciences, University of Manitoba, Winnipeg, Manitoba, Canada.; 7 Center of Research Excellence in Allergy and Immunology, Research Department, Faculty of Medicine Siriraj Hospital, Mahidol University, Bangkok, Thailand.

**Keywords:** Immunology, Neuroscience, Pain

## Abstract

Acute severe joint pain is a major symptom in gouty arthritis (GA), and its adequate treatment represents an unmet medical need. Mrgprb2, a specific mast cell receptor, has been implicated in the generation of chronic pain by mobilizing mast cell degranulation, yet its significance in GA pain and joint inflammation is still not well defined. Here, we found that Mrgprb2 was expressed in mouse synovial mast cells. In a murine model of GA, acute blockade or genetic deletion of Mrgprb2 significantly attenuated arthritis pain and hyperexcitability of joint nociceptors with significant reductions in innate immune cell recruitment in the synovium. Under naive conditions, activation of synovial Mrgprb2 was sufficient to excite peripheral terminals of joint nociceptors to induce acute joint hypernociception via the mobilization of mast cell degranulation. Additionally, the level of the neuropeptide substance P (SP) was elevated in the synovium of GA model mice. Using humanized MRGPRX2-knockin mice, we revealed that SP contributed to joint pain and inflammation by activating mast cells through Mrgprb2/MRGPRX2. These findings suggest that synovial mast cell–expressed Mrgprb2/MRGPRX2 merits consideration as a key neuroimmune player and a potential therapeutic target for treating GA pain and joint inflammation.

## Abstract 2

This study illuminates a neuroimmne mechanism that MRGPRX2 signaling in synovial mast cells drives gout pain and joint inflammation.

## Graphical abstract

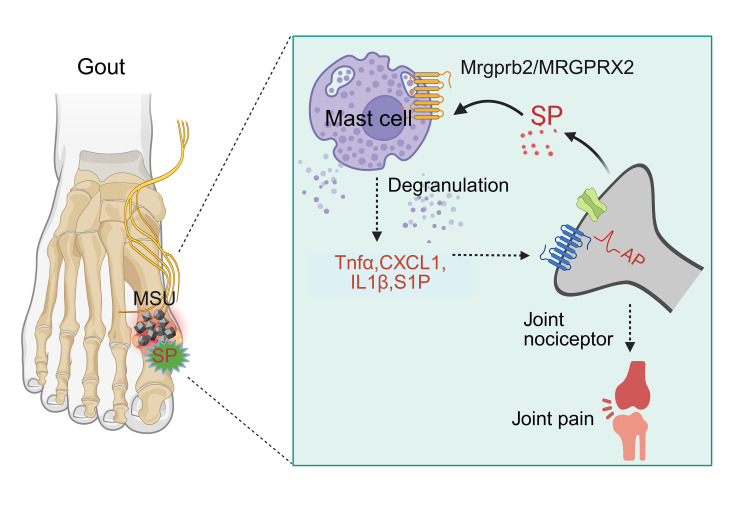

## Introduction

Gouty arthritis (GA) is a common inflammatory joint disease triggered by the accumulation of monosodium urate (MSU) crystals in the joint, affecting up of 6.8% of adults worldwide (1, 2). Severe joint pain is a major clinical symptom in patients with GA, and represents a huge health burden (3, 4). GA is characterized by hyperuricemia and joint inflammation, both of which are thought be critical drivers of GA pain (3, 4). Accordingly, most current therapies for acute gout attacks are aimed at lowering uric acid levels and reducing joint inflammation (3). Yet many of these managements exhibit low efficacy and/or severe side effects (3). Thus, identification of new therapeutic targets for gout pain is urgently needed.

Mast cells (MCs) are one key component of the innate immune system, and have been implicated in the pathogenesis of GA (5, 6). Increased numbers of MCs were observed in the inflamed synovium in the setting of GA (5). Synovial MCs can serve as a reservoir of proinflammatory mediators in the context of GA. Upon activation, MCs release a cascade of cytokines and chemokines, further facilitating the subsequent immune cell recruitment and sensitization of peripheral nerve fibers (6). MCs express various surface receptors, including Fc receptors for IgE, G protein–coupled receptors (GPCRs), and cytokine and chemokine receptors (6, 7). Thus, MCs can be activated in two ways: by IgE-mediated classical allergic reactions and by IgE-independent pseudoallergic reactions (8, 9). However, the exact mechanism of synovial MC activation during GA still remains largely unexplored.

Mas-related GPCRs (Mrgprs) are a family of GPCRs present on innate immune cells and primary sensory neurons, which mediate various noxious sensations and function as pain and itch receptors (10, 11). Mrgprb2 in mice and its ortholog, human MRGPRX2, are selectively expressed on connective tissue MCs (CTMCs), including human synovial MCs (12, 13). Yet it is still unclear whether Mrgprb2 is expressed in mouse synovium and what is the role of synovial Mrgprb2/MRGPRX2 during GA. Both Mrgprb2 and MRGPRX2 act as a critical regulator of MC activation and are activated by a variety of basic secretagogues through a non-IgE-dependent mechanism (12, 14). Mrgprb2/MRGPRX2 activation of MCs has been involved in the pathogenesis of chronic pain in various pain models, including migraine, chemotherapy-induced pain, postoperative pain, alcohol withdrawal–associated headache, and endometriosis pain (15–19). However, it is unclear whether activation of synovial MCs via Mrgprb2/MRGPRX2 is necessary and sufficient to drive the development of GA pain and joint inflammation.

As potent innate immune effector cells, MCs are essential to the communication between the immune and peripheral nervous systems (20, 21). MCs are often found to be in close contact with peripheral nerve terminals in the synovium (22). This unique anatomical feature enables MCs to behave as the first innate immune cell in response to peripheral sensory nerve activation (23). Mrgprb2/MRGPRX2 functions as a key modulator of MC–sensory neuron crosstalk and contributes to inflammatory pain and allergic itch (9, 15). More importantly, several neuropeptides were recently identified as endogenous agonists of Mrgprb2/MRGPRX2 to activate MCs, including substance P (SP) and pituitary adenylated cyclase–activated peptide (PACAP) (15, 18). Furthermore, SP and PACAP can favor MC recruitment to sensory nerve fibers via Mrgprb2, leading to peripheral sensitization and the associated postoperative pain or migraine (9, 15, 24). However, no studies have directly addressed whether and how Mrgprb2/MRGPRX2 signaling is involved in synovial MC–neuron crosstalk in the context of GA.

Here, we identified that Mrgprb2 was expressed on mouse synovial MCs. Furthermore, Mrgprb2/MRGPRX2-mediated MC activation contributed to joint pain and inflammation under the condition of GA. In addition, SP likely acts as an endogenous agonist of Mrgprb2/MRGPRX2 and drives joint pain and inflammatory responses via the activation of MCs during GA.

## Results

### Mrgprb2-expressing synovial MCs are activated during GA.

**Figure 1:**
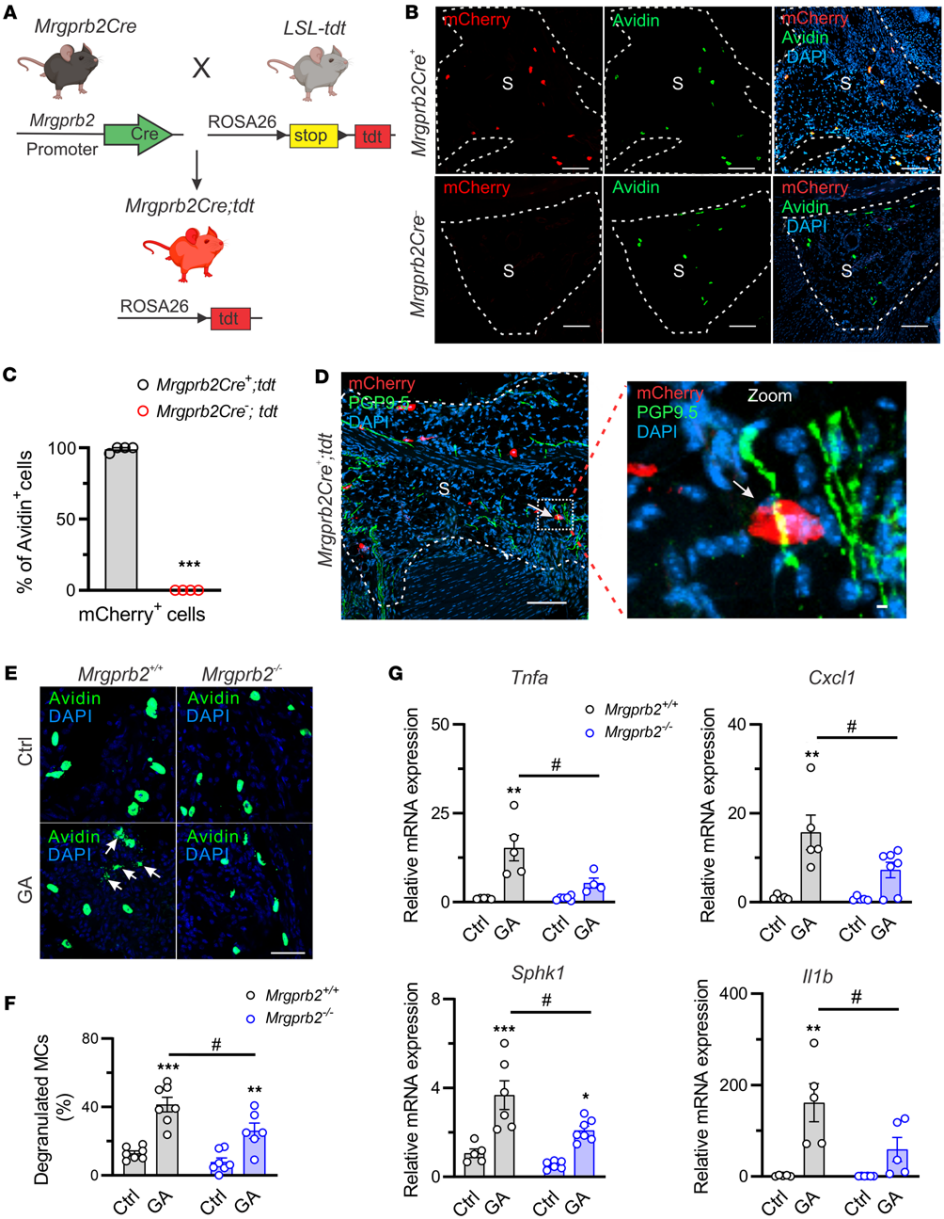
Mrgprb2 is expressed in synovial MCs and is activated following GA. (**A**) Strategy for the generation of *Mrgprb2Cre;tdt* reporter mice. tdt, tdTomato. Figure created in BioRender (Qu L, 2026, https://BioRender.com/jh5fc7d). (**B**) IHC images of knee sections showing tdt^+^ cells (red; stained with mCherry) in the synovium of *Mrgprb2Cre^+^* mice but not *Mrgprb2Cre^–^* mice. All tdt^+^ cells in mouse synovium were also stained with avidin (green), a pan marker for MCs. S, synovium. Scale bars: 100 μm. (**C**) Summary of the colocalization of tdt^+^ cells and avidin-stained MCs. *n* = 4 mice per group; ***P < 0.001 vs. *Mrgprb2Cre^+^;tdt* mice; unpaired 2-tailed Student’s *t* test. (**D**) Immunostaining showing the spatial distribution of tdt^+^ labeled MCs (red) and joint nerve fiber stained with PGP9.5 (green). Arrows indicate MCs that are in close proximity to joint nerve fibers. S, synovium. Scale bars: 100 μm. (**E**) Representative images of knee joint sections stained with avidin in control (Ctrl) and GA groups of each genotype. Arrows show degranulated MCs. Scale bar: 50 μm. (**F**) Percentage of degranulated MCs in the synovium of *Mrgprb2^+/+^* and *Mrgprb2^–/–^* mice on day 1 after GA. *n* = 6–7 mice per group; **P < 0.01, ***P < 0.001 vs. Ctrl; ^#^*P* < 0.05 vs. *Mrgprb2^+/+^* mice; 2-way ANOVA for repeated measures followed by Bonferroni’s post hoc test. (**G**) Quantitative PCR assay showing inflammation-related gene expression, including *Tnfa*, *Cxcl1*, *Sphk1*, and *Il1b* on day 1 after PBS (Ctrl) and MSU challenge (GA). *n* = 4–7 mice per group; *P < 0.05, **P < 0.01, ***P < 0.001 vs. Ctrl; ^#^*P* < 0.05 vs. *Mrgprb2^+/+^* mice; 2-way ANOVA (repeated measures) with Bonferroni’s post hoc test.

We first sought to investigate whether Mrgprb2 is present in mouse synovial MCs. Since there are no commercially available anti-Mrgprb2 antibodies specific for reliable IHC staining in mouse tissues, we utilized *Mrgprb2Cre;tdTomato* (tdt) mice, which express tdt fluorescent protein specifically in cells expressing Mrgprb2 (Figure 1A) (12, 15). We detected tdt^+^ cells in the synovium of *Mrgprb2Cre^+^* mice but not in that of *Mrgprb2Cre^–^* controls (Figure 1, B and C). Furthermore, using the pan-MC marker avidin, we observed tdt signals in all avidin-stained cells in mouse synovium, confirming that almost all synovial MCs express Mrgprb2 (Figure 1, B and C). Given that MCs were observed in close proximity to peripheral nerve endings in the skin (15), we next asked whether this unique anatomical feature is also present in the synovium. Similar to previous observations in the skin, a portion of Mrgpb2-expressing MCs were near PGP9.5-labeled nerve fibers in mouse synovium, which provides an anatomical basis for synovial MC-neuron communications (Figure 1D).

To assess whether Mrgprb2 is capable of modulating the activity of synovial MCs, we monitored small vesicle release near MCs as a marker of degranulation. We observed that synovial MCs of both *Mrgprb2^+/+^* and *Mrgprb2^–/–^* mice exhibited very low activation and showed no significant differences in degranulation between genotypes in the control group (Figure 1, E and F). Deleting *Mrgprb2* significantly decreased the proportion of degranulated MCs in the synovium in comparison with *Mrgprb2^+/+^* mice on day 1 following GA (Figure 1, E and F). We additionally assessed the mRNA expression of various proinflammatory mediators potentially released by MCs in the synovium as a biomarker of MC activity in the setting of GA. Among all the genes tested, GA resulted in a significant upregulation of *Tnfa*, *Cxcl1*, *Sphk1*, and *Il1b* in the synovium of *Mrgprb2^+/+^* mice on day 1 after GA (Figure 1G). However, such effects were diminished in *Mrgprb2^–/–^* mice (Figure 1G). Taken together, genetic deletion of *Mrgprb2* thus results in reduced synovial MC degranulation and diminished cytokine production, indicating that Mrgprb2 functions as a critical receptor for synovial MC activation during GA.

### Genetic deletion of Mrgprb2 attenuates joint pain and inflammation in acute gout model.

**Figure 2:**
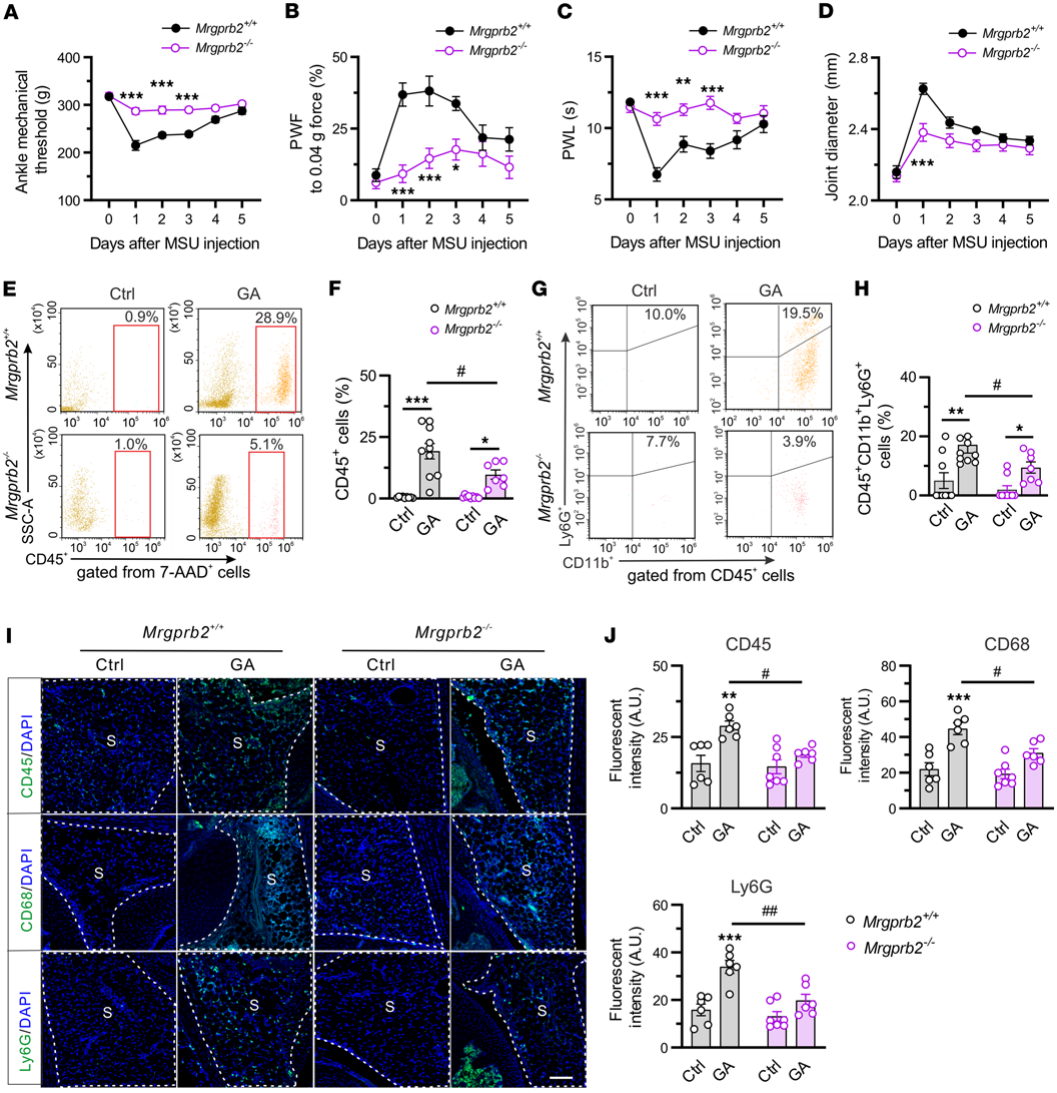
Deleting *Mrgprb2* alleviates GA pain and joint inflammation. (**A**–**D**) Mechanical threshold in the hind ankle (**A**), paw withdrawal frequency (PWF) in response to 0.04*g* force in the hind paw (**B**), paw withdrawal latency (PWL) to radiant heat in the hind paw (**C**), and ankle joint diameter (**D**) in *Mrgprb2^+/+^* (*n* = 10) and *Mrgprb2^–/–^* (*n* = 11) mice over the time course of GA. *P < 0.05, **P < 0.01, ***P < 0.001 vs. *Mrgprb2^+/+^*; 2-way ANOVA (repeated measures) followed by Bonferroni’s correction. (**E**–**H**) Flow cytometry analysis of CD45^+^ (**E**), CD11b^+^, and Ly6G^+^ cells (**G**) in the synovium of *Mrgprb2^+/+^* and *Mrgprb2^–/–^* mice 1 day after vehicle control (Ctrl) and MSU (GA) challenge. Summary of the percentage of CD45^+^ cells (**F**) and CD45^+^CD11b^+^Ly6G^+^ neutrophils (**H**). *n* = 7–10 mice per group; *P < 0.05, **P < 0.01, ***P < 0.001 vs. Ctrl; ^#^*P* < 0.05 vs. *Mrgprb2^+/+^*; repeated 2-way ANOVA followed by Bonferroni’s post hoc test. (**I**) Immunofluorescence images of knee joint tissues from *Mrgprb2^+/+^* and *Mrgprb2^–/–^* mice 1 day after vehicle and MSU challenge, stained for CD45, CD68, and Ly6G. S, synovium. Scale bar: 100 μm. (**J**) Data analysis revealed a remarkable increase in all tested immune cell markers in the synovium following GA. Such effects were diminished in *Mrgprb2^–/–^* mice. *n* = 6 mice per group; **P < 0.01, ***P < 0.001 vs. Ctrl; ^#^*P* < 0.05, ^##^*P* < 0.01 vs. *Mrgprb2^+/+^*; 2-way ANOVA (repeated measures) followed by Bonferroni’s post hoc test.

To evaluate whether Mrgprb2 contributes to GA pain, we compared pain-like behaviors between *Mrgprb2^+/+^* and *Mrgprb2^–/–^* mice in a mouse GA model elicited by injection of MSU into hind ankle joints. On day 1 after MSU injection, both *Mrgprb2^+/+^* and *Mrgprb2^–/–^* mice developed primary mechanical hyperalgesia compared with baseline (Figure 2A). However, such effects were significantly diminished in *Mrgprb2^–/–^* animals compared with wild-type (WT) controls. Similarly, *Mrgprb2^–/–^* mice exhibited less secondary mechanical and thermal hyperalgesia in the hind paw, and joint swelling over the course of acute gout, compared with the WT group (Figure 2, B–D). Both pain hypersensitivity and joint inflammation peaked at approximately day 1 after injection of MSU into mice and returned to baseline at day 5. Yet we did not observe any sex differences in analgesic effects of *Mrgprb2* deletion under the condition of GA (Supplemental Figure 1; supplemental material available online with this article; https://doi.org/10.1172/jci.insight.201781DS1). Next, we asked whether our findings are generalized to other inflammatory arthritis models. CFA-induced arthritis is known as another animal model of inflammatory arthritis (25, 26). As expected, similar findings were observed in a CFA-induced inflammatory arthritis model, in which both mechanical and thermal hyperalgesia was attenuated in *Mrgprb2^–/–^* mice (Supplemental Figure 2).

Considering MCs as a crucial driver of inflammation in the setting of acute gout (6), we further determined whether the apparent analgesic effects of *Mrgprb2* deletion were due to a possible attenuation of joint inflammation. Neutrophil infiltration into joints is a key feature of acute GA and is of importance to the pathogenesis of joint inflammation and GA pain (27, 28). Given that MCs are critical for the recruitment of neutrophils in other injuries, we mainly focus on neutrophil infiltration during GA. Flow cytometry assay revealed that the leukocyte population (labeled with CD45) was significantly increased in the synovium of both *Mrgprb2^+/+^* and *Mrgprb2^–/–^* mice on day 1 following gout arthritis, but the extent of an increase with CD45^+^ cells in *Mrgprb2^–/–^* mice was much less than in WT animals (Figure 2, E and F). Furthermore, we observed a similar immune cell recruitment pattern in neutrophils (Ly6G^+^CD11b^+^) (Figure 2, G and H). In addition, joint IHC analysis showed that all assayed immune cell markers (CD45, Ly6G, CD68) were increased on day 1 after GA induction (Figure 2, I and J). However, the observed synovial immune cell infiltration was much less in *Mrgprb2^–/–^* mice (Figure 2, I and J). Notably, both flow cytometry assay and joint IHC staining did not detect any deficits in MC, neutrophil, and monocyte populations in naive *Mrgprb2^–/–^* mice as reported previously (Figure 2, E–H, and Supplemental Figure 3) (15). These results highlight a key role of Mrgprb2 in arthritis pain and joint inflammation during GA.

### Acute pharmacological inhibition of Mrgprb2 reverses GA pain and joint inflammation.

**Figure 3:**
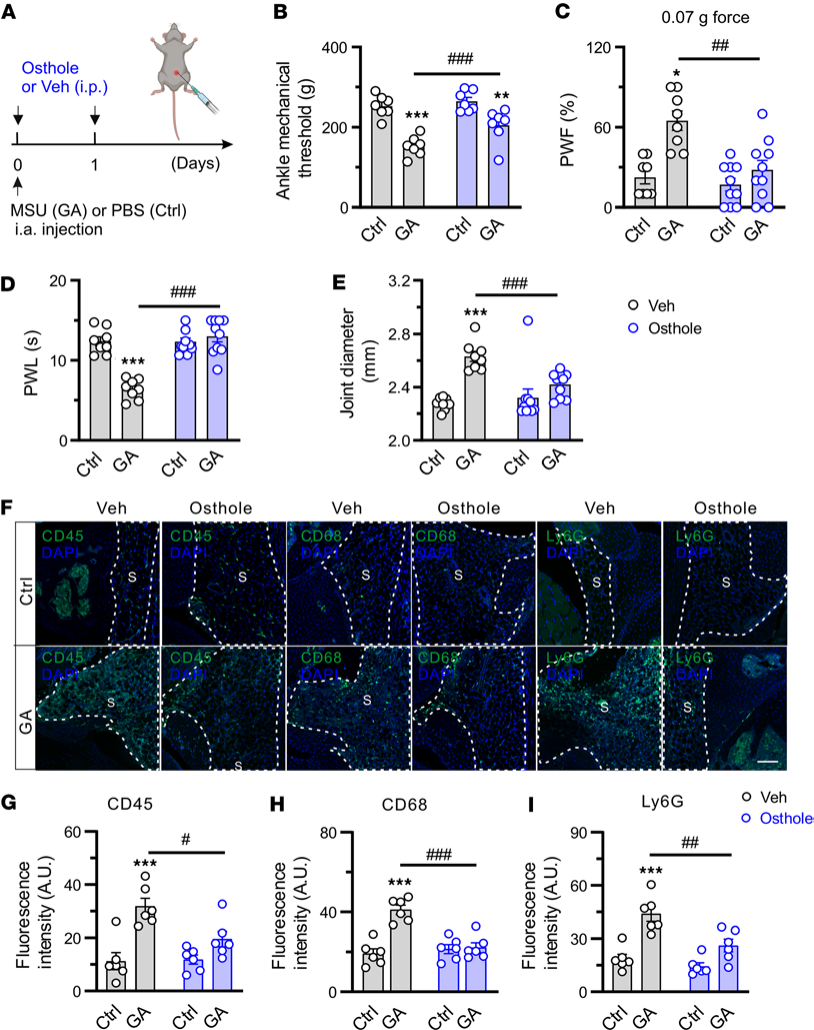
Pharmacological inhibition of Mrgprb2 reverses GA pain and joint inflammation. (**A**) Experimental schematic showing once-daily i.p. injection with osthole (3 mg, 20 μL) or vehicle (Veh) (1% DMSO; 20 μL) on days 0 and 1 in mice challenged with PBS (Ctrl) or MSU (GA). Pain-related behaviors were measured 1 hour after injection. Figure created in BioRender (Qu L, 2026, https://BioRender.com/0i7869s). (**B**–**E**) The effect of repeated daily i.p. injection of osthole or vehicle on mechanical threshold in the ankle (**B**), paw withdrawal frequency (PWF) responding to 0.07*g* force (**C**), paw withdrawal latency (PWL) (**D**), and ankle diameter (**E**) in GA model mice. *n* = 7 mice per group; *P < 0.05, **P < 0.01, ***P < 0.001 vs. Ctrl; ^##^*P* < 0.01, ^###^*P* < 0.001 vs. Veh; repeated 2-way ANOVA with Bonferroni’s post hoc test. (**F**) Images of knee joints taken 1 hour after the second i.p. injection with vehicle or osthole and stained with CD45, CD68, or Ly6G. S, synovium. Scale bar: 100 μm. (**G**–**I**) Quantification showed that osthole significantly reduced the infiltration of CD45^+^ (**G**), CD68^+^ (**H**), and Ly6G^+^ (**I**) cells into the inflamed synovium of GA mice. *n* = 6 mice per group; ***P < 0.001 vs. Ctrl; ^#^*P* < 0.05, ^##^*P* < 0.01, ^###^*P* < 0.001 vs. Veh; 2-way ANOVA (repeated measures) with Bonferroni’s post hoc test.

To avoid potential confounding effects of genetic deletion of *Mrgprb2*, we examined whether pharmacological inhibition of Mrgprb2 could alleviate GA pain and joint inflammation. Osthole, an important natural coumarin present in the fruits of the *Cnidium monnieri*, is a known Mrgprb2/MRGPRX2 blocker (29). We administered osthole or vehicle i.p. into WT mice once daily on days 0 and 1 after acute gout induction. Both mechanical and thermal hyperalgesia was evaluated within 2 hours after each injection (Figure 3A). In GA mice with established joint pain hypersensitivity, systemic administration of osthole rapidly alleviated mechanical hyperalgesia in the inflamed ankle, and both mechanical and thermal hyperalgesia in the ipsilateral hind paw (Figure 3, B–E). In addition, mice treated with osthole exhibited less joint swelling than those subjected to vehicle controls (Figure 3E). Next, we asked whether the analgesic effects of osthole are secondary to a decrease of immune cell infiltration into the synovium. Systemic administration of osthole significantly decreased the expression of CD45, Ly6G, and CD68 in the synovium of GA mice, compared with vehicle (Figure 3, F–I). These data further point to a critical role of Mrgprb2 in the development of GA pain and joint inflammation.

### Genetic deletion of Mrgprb2 reduces hyperexcitability of joint sensory neurons following acute gout.

**Figure 4:**
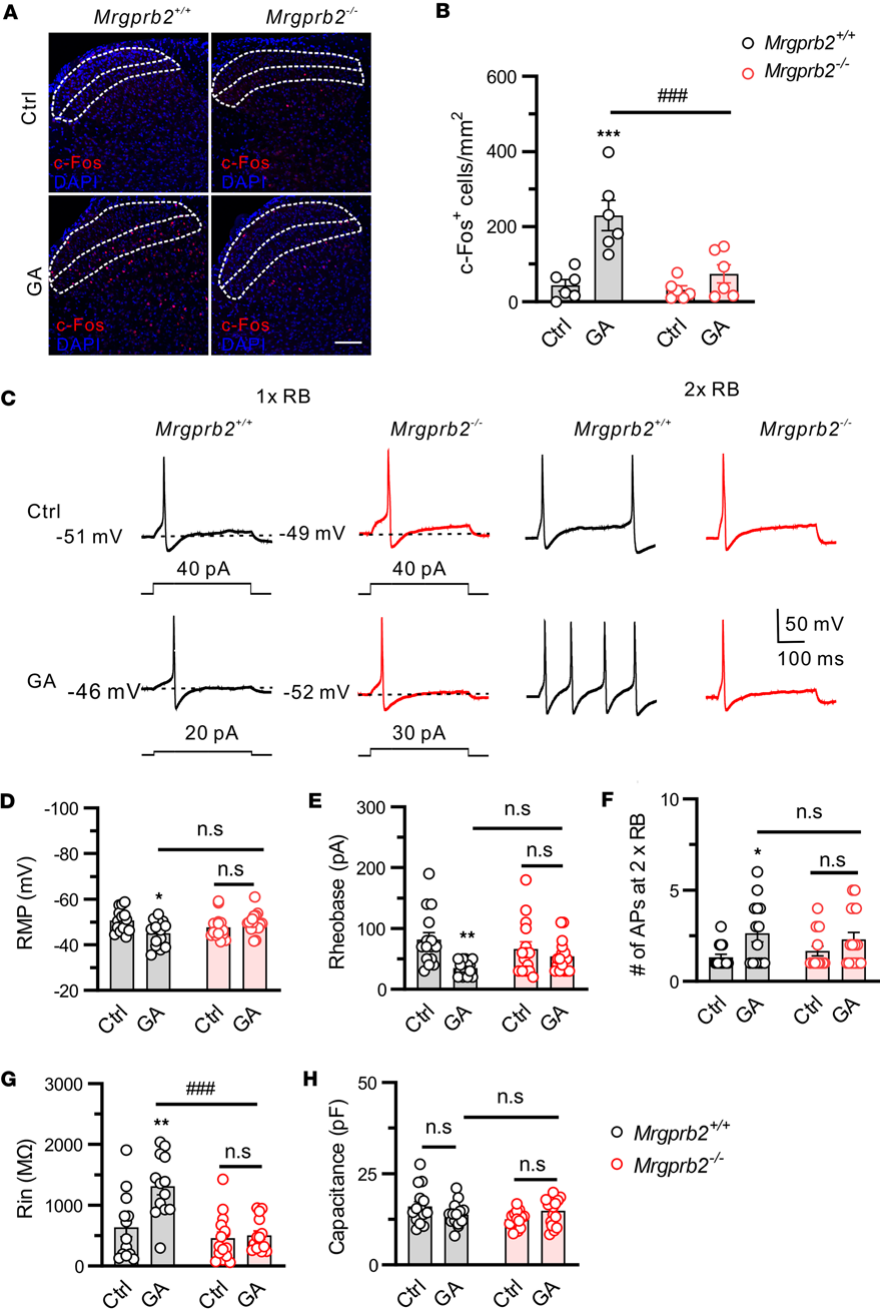
Mrgprb2 contributes to hyperexcitability of joint nociceptors following GA. (**A**) Immunostaining showing c-Fos expression in the spinal cord of *Mrgprb2^+/+^* and *Mrgprb2^–/–^* mice 1 day after vehicle control (Ctrl) and MSU (GA) challenge. Scale bar: 100 μm. (**B**) Quantification revealed an increase in c-Fos^+^ cells in the spinal cord dorsal horn layer I–II of *Mrgprb2^+/+^* GA model mice. No such effects were observed in *Mrgprb2^–/–^* mice with GA. *n* = 6 mice per group; ***P < 0.001 vs. Ctrl; ^###^*P* < 0.001 vs. *Mrgprb2^+/+^*; 2-way ANOVA (repeated measures) followed by Bonferroni’s correction. (**C**) Representative traces of action potentials (APs) evoked by rheobase (RB) and twice (2×) RB in DiI-labeled joint nociceptors from *Mrgprb2^+/+^* and *Mrgprb2^–/–^* mice 1 day after vehicle control (Ctrl) and MSU (GA) challenge. (**D**–**H**) Joint nociceptors of *Mrgprb2^+/+^* mice displayed a more depolarized resting membrane potential (RMP; **D**), lower mean rheobase (**E**), larger number of APs at twice rheobase (**F**), and increased input resistance (Rin; **G**) on day 1 after GA induction. Genetic deletion of *Mrgprb2* prevented these changes. No significant difference in cell capacitance was observed between groups (**H**). *n* = 14–17 neurons; *P < 0.05, **P < 0.01 vs. Ctrl; ^###^*P* < 0.001vs. *Mrgprb2^+/+^*; 2-way ANOVA (repeated measures) followed by Bonferroni’s correction.

Sensitization of joint nociceptors represents an important neuronal mechanism of arthritis pain (30). We next evaluated whether Mrgprb2 is involved in peripheral sensitization of joint nociceptors in the GA model. Neuronal activation was measured in the superficial layer of the dorsal horn of the spinal cord where nociceptive primary afferents normally terminate via the induction of c-Fos expression (31, 32). On day 1 after GA induction, the percentage of c-Fos^+^ cells in the superficial layer of the dorsal horn of the spinal cord was significantly increased in *Mrgprb2^+/+^* mice with GA, compared with control animals (Figure 4, A and B). However, such effects were diminished in *Mrgprb2^–/–^* mice (Figure 4, A and B). To further assess whether Mrgprb2 contributes to hyperexcitability of joint nociceptors in the context of acute gout, we performed patch clamp recordings on dissociated DiI-labeled joint-innervating dorsal root ganglion (DRG) neurons of both *Mrgprb2^+/+^* and *Mrgprb2^–/–^* mice following GA. On day 1 after gout induction, joint sensory neurons recorded from WT animals exhibited depolarized resting membrane potential, lower rheobase, the larger mean number of action potentials evoked by twice rheobase, and increased input resistance compared with control animals (Figure 4, C–G). However, no such effects were observed in joint nociceptors from *Mrgprb2^–/–^* mice. There were no significant changes with membrane capacitance for either genotype following GA (Figure 4H). These data suggest that Mrgprb2 contributes to hyperexcitability of joint nociceptors following GA.

### Activation of Mrgprb2 expressed by synovial MCs drives acute joint hypernociception and inflammation in naive mice.

**Figure 5:**
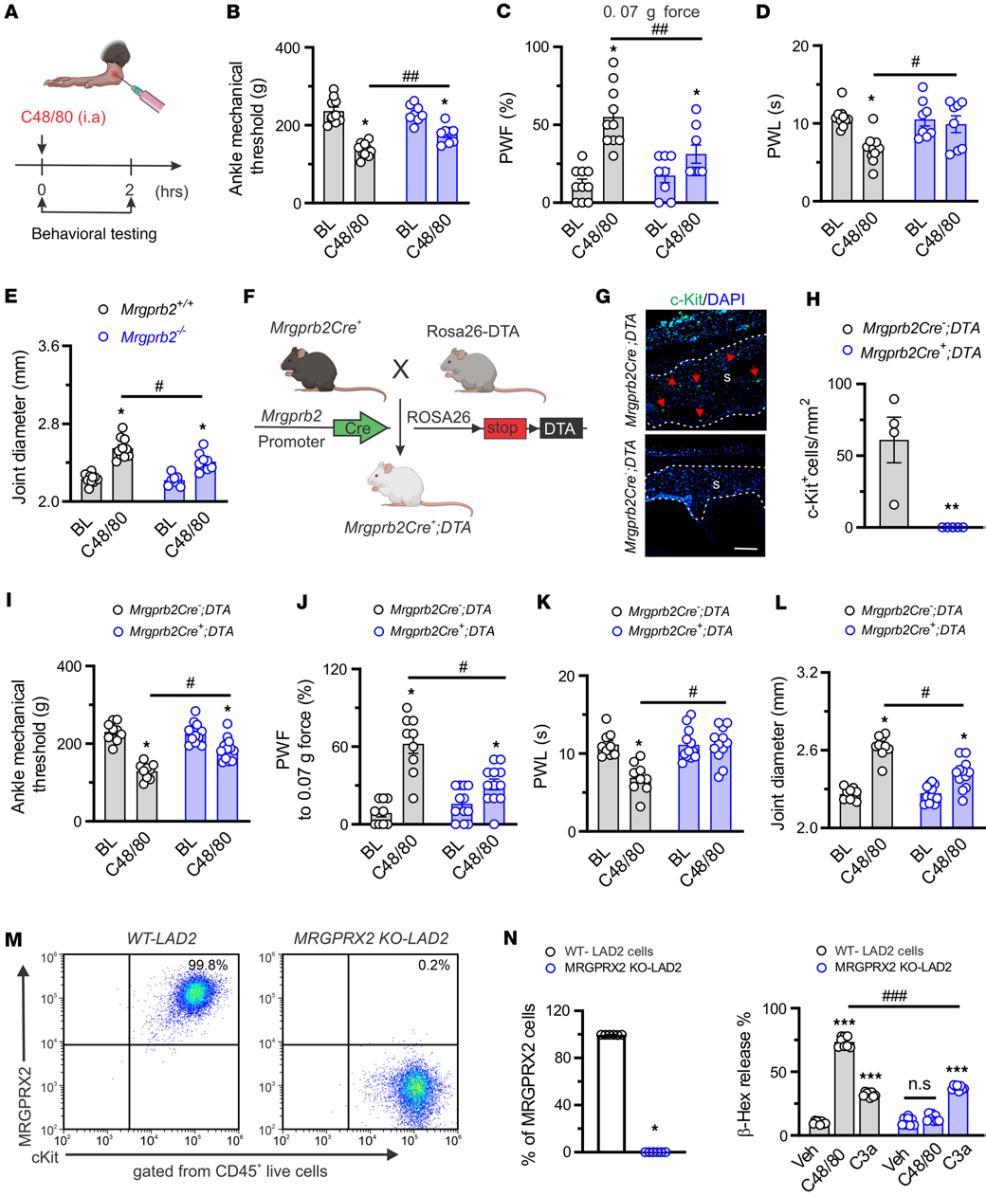
Activation of synovial Mrgprb2 by C48/80 evokes behavioral signs of joint hypernociception. (**A**) Schematic illustration of intra-articular (i.a.) administration protocol of C48/80 (1.5 μg, 10 μL). Pain-related behaviors were assessed within 2 hours after injection. Figure created in BioRender (Qu L, 2026, https://BioRender.com/snnmb6q). (**B**–**E**) Changes of mechanical threshold in the ankle (**B**), paw withdrawal frequency (PWF) responding to 0.07*g* force in the hind paw (**C**), paw withdrawal latency (PWL) to radiant heat in the hind paw (**D**), and joint diameter (**E**) following i.a. injection of C48/80 in *Mrgprb2^+/+^* (*n* = 10) and *Mrgprb2^–/–^* (*n* = 9) mice. *P < 0.05 vs. baseline (BL); ^#^*P* < 0.05, ^##^*P* < 0.01 vs. *Mrgprb2^+/+^*; 2-way ANOVA (repeated measures) followed by Bonferroni’s correction. (**F**) Generation of *Mrgprb2Cre DTA* mouse line. Figure created in BioRender (Qu L, 2026, https://BioRender.com/t0h8dej). (**G**) Representative IHC images of knee joint sections stained for c-Kit (green) in *Mrgprb2Cre^+^;DTA* and *Cre^–^* littermates. Red arrows show c-Kit^+^ cells. S, synovium. Scale bar: 200 μm. (**H**) Quantification showed a loss of MCs in the synovium of *Mrgprb2Cre^+^;DTA* mice but not *Cre^–^* littermate controls. *n* = 4 mice per group; **P < 0.01 vs. *Mrgprb2Cre^+^;DTA*; unpaired 2-tailed Student’s *t* test. (**I**–**L**) *Mrgprb2Cre^+^;DTA* mice exhibited higher mechanical threshold in the ankle (**I**), lower PWF in response to 0.07*g* force (**J**), longer PWL in the hind paw (**K**), and smaller ankle diameter (**L**) following i.a. injection of C48/80 compared with *Cre^–^* littermates. *P < 0.05 vs. BL; ^#^*P* < 0.05 vs. *Mrgprb2Cre^–^ DTA*; 2-way ANOVA (repeated measures) followed by Bonferroni’s correction. (**M**) Representative flow cytometric profiles of MRGPRX2 expression (CD45^+^c-Kit^+^MRGPRX2^+^) in WT and MRGPRX2-knockout LAD2 (MRGPRX2-KO-LAD2) cells. (**N**) Quantification showed loss of MRGPRX2 expression in MRGPRX2-KO-LAD2 cells compared with WT controls. *n* = 6 biological repeats per group; *P < 0.001 vs. WT; unpaired 2-tailed Student’s *t* test. (**O**) C48/80- and C3a-induced β-hexosaminidase (β-hex) release in WT and MRGPRX2-KO-LAD2 cells. *n* = 8 biological repeats per group; ***P < 0.001 vs. Veh; ^###^*P* < 0.001 vs. WT-LAD2 cells; 2-way ANOVA (repeated measures) followed by Bonferroni’s correction.

Compound 48/80 (C48/80) represents a classical MC agonist and canonical basic secretagogue through Mrgprb2 (12). Next, we sought to explore a potential role of Mrgprb2-expressing synovial MCs in joint pain hypersensitivity in vivo using C48/80 as an agonist (Figure 5A). Injection of C48/80 to the right ankle cavity lowered mechanical threshold in the hind ankle and increased frequency of hind paw withdrawal in response to mechanical stimulation in both *Mrgprb2^+/+^* and *Mrgprb2^–/–^* mice (Figure 5, B and C). However, such effects were diminished in *Mrgprb2^–/–^* mice. In addition, *Mrgprb2^–/–^* mice exhibited less secondary thermal hyperalgesia and less joint swelling evoked by C48/80 than WT controls (Figure 5, D and E). To further evaluate the contribution of Mrgprb2 to acute joint hypernociception, we genetically ablated MCs by mating *Mrgprb2Cre* mice with DTA mice (Figure 5F). IHC assay confirmed a loss of synovial MCs in *Mrgprb2Cre^+^;DTA* mice but not in *Mrgprb2Cre^–^* littermates (Figure 5, G and H). No significant differences in basal mechanical and thermal sensitivity at the hind ankle or hind paw skin were observed between genotypes (Figure 5, I–L). However, C48/80-evoked nocifensive behaviors and joint swelling were alleviated in *Mrgprb2Cre^+^* mice compared with *Mrgprb2Cre^–^* littermates (Figure 5, I–L). These results suggest that Mrgrpb2 activation of synovial MCs is sufficient to trigger articular hypernociception. To further determine whether C48/80 can activate human MCs through MRGPRX2, we compared the effects of C48/80 on the degranulation of WT LAD2 cells, a human MC line, and LAD2 cells in which MRGPRX2 was genetically deleted (MRGPRX2-KO-LAD2). The loss of MRGPRX2 expression in LAD2 cells was confirmed by flow cytometry (Figure 5, M and N). Application of C48/80 elicited the release of β-hexosaminidase from WT LAD2 cells but not MRGPRX2-KO-LAD2 cells (Figure 5O). Notably, deleting MRGPRX2 did not impair complement 3a–induced (C3a-induced) MC degranulation in LAD2 cells (Figure 5O).

**Figure 6:**
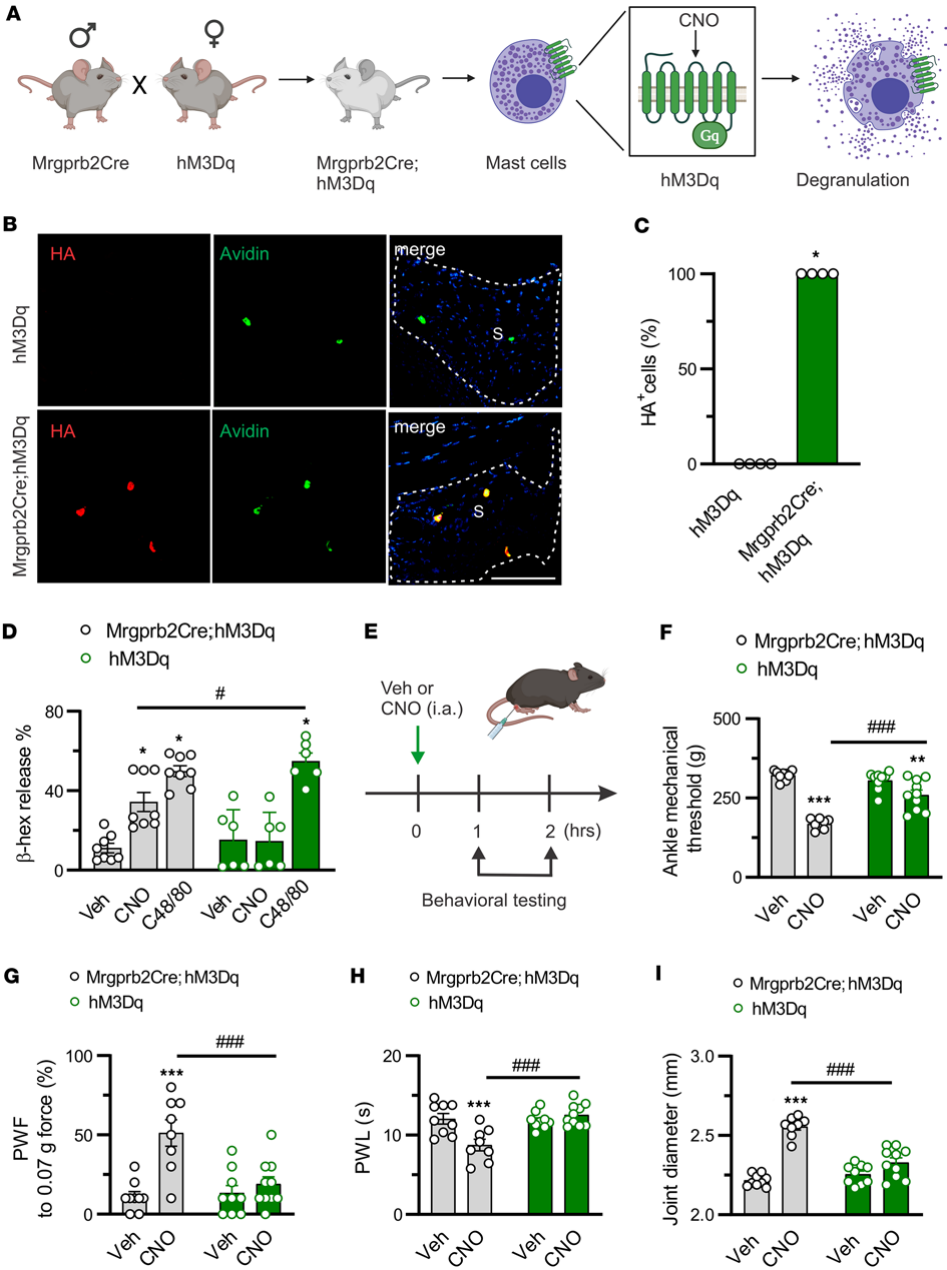
Chemogenetic activation of synovial Mrgprb2-expressing MCs drives joint pain hypersensitivity. (**A**) Schematic of the *Mrgprb2Cre;hM3Dq* mouse line generated by mating of *Mrgprb2Cre* mice with a Rosa26-LSL-hM3Dq mouse line. Figure created in BioRender (Qu L, 2026, https://BioRender.com/cdliw1r). (**B**) Immunofluorescence images of knee joint sections showed the expression of hM3Dq (stained with anti-HA tag; red) in synovial MCs (stained with avidin; green) of *Mrgprb2Cre^+^* mice but not *Cre^–^* littermates. S, synovium. Scale bar: 200 μm. (**C**) Quantitative analysis of percentage overlap. *n* = 4 mice per group; *P < 0.05 vs. *hM3Dq*; unpaired 2-tailed Student’s *t* test. (**D**) CNO- and C48/80-induced β-hexosaminidase (β-hex) release from PDMCs isolated from *Mrgprb2Cre;hM3Dq* mice (*n* = 7) and *Cre^–^* littermates (*n* = 6). *P < 0.05 vs. Veh (saline); ^#^*P* < 0.05 vs. *hM3Dq*; 2-way ANOVA (repeated measures) followed by Bonferroni’s correction. (**E**) Intra-articular administration protocol of CNO (20 μg, 10 μL) and vehicle (Veh; saline, 10 μL). Pain-like behaviors were evaluated within 2 hours after injection. Figure created in BioRender (Qu L, 2026, https://BioRender.com/dyysb9x). (**F**–**I**) *Mrgprb2Cre;hM3Dq* mice treated with CNO exhibited lower mechanical threshold in the ankle (**F**), higher paw withdrawal frequency (PWF) responding to 0.07*g* force (**G**), prolonged paw withdrawal latency (PWL) to radiant heat in the hind paw (**H**), and reduced ankle diameter (**I**) compared with *Cre^–^* littermates with CNO treatment. *n* = 8–10 mice per group; **P < 0.01, ***P < 0.001 vs. Veh; ^###^*P* < 0.001 vs. *hM3Dq*; 2-way ANOVA (repeated measures) followed by Bonferroni’s correction.

C48/80 was shown to act directly on neurons besides MCs (33). To circumvent this confounding effect, we used designer receptor exclusively activated by designer drugs (DREADD) strategy to chemogenetically activate Mrgprb2-lineage MCs. This was achieved by mating of *Mrgprb2Cre* mice with a Cre-dependent Rosa26-LSL-hM3Dq mouse line (*Mrgprb2Cre;hM3Dq*) in which hM3Dq, a GPCR, was specifically expressed in Mrgprb2-lineage MCs (Figure 6A). Thus, this specific population of MCs can be activated through hM3Dq by the synthetic ligand clozapine *N*-oxide (CNO), a compound that cannot activate endogenous GPCRs (Figure 6A) (34, 35). Anatomical confirmation of the resulting expression of hM3Dq in synovial MCs of *Mrgprb2Cre^+^* mice but not *Mrgprb2Cre^–^* littermates was achieved by staining of the synovium with anti-HA (for hM3Dq) and avidin (for MCs) (Figure 6, B and C). To determine whether CNO is sufficient to activate MCs, we compared the effects of CNO on degranulation of peritoneal-derived mast cells (PDMCs) obtained from *Mrgprb2Cre;hM3Dq* mice and *Cre^–^* littermates. Application of CNO induced the release of β-hexosaminidase in PDMCs from *Mrgprb2Cre^+^* mice but not from *Mrgprb2Cre^–^* littermates (Figure 6D). Yet C48/80-induced MC degranulation was not impaired in either genotype (Figure 6D). Local injection of CNO into the right hind ankle of mice induced robust primary mechanical hyperalgesia in the ankle, secondary mechanical and thermal hyperalgesia in the hind paws, and joint swelling within 2 hours in *Mrgprb2Cre^+^;hM3Dq* mice (Figure 6, E–I). Yet no such effects were observed in *Mrgprb2Cre^–^* littermates (Figure 6, E–I). Collectively, these results suggest that Mrgprb2 activation of synovial MCs is sufficient to drive acute joint pain hypersensitivity and inflammation in the naive state.

### Mrgprb2 activation of synovial MCs activates peripheral terminals of joint sensory neurons in naive mice.

**Figure 7:**
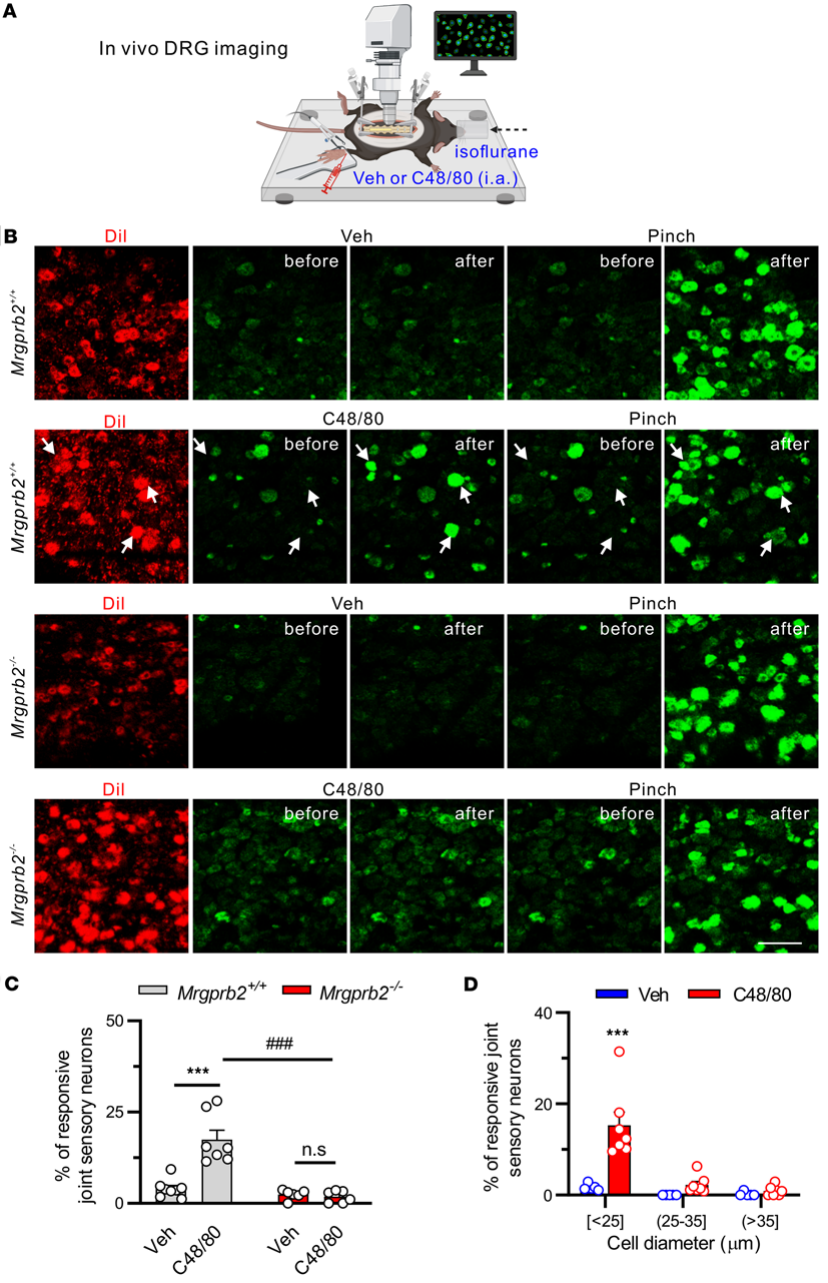
Activation of synovial Mrgprb2 by C48/80 activates peripheral terminals of joint sensory neurons in vivo. (**A**) Schematic of in vivo DRG imaging in anesthetized mice. Figure created in BioRender (Qu L, 2026, https://BioRender.com/tvek5ah). (**B**) Left column: Representative DiI fluorescence (red) in cell bodies of L4 DRG in *Pirt-Cre-GCamp6* mice that were retrogradely labeled with ankle joint injection of DiI into either *Mrgprb2^+/+^* or *Mrgprb2^–/–^* mice (2 mg/mL, 10 μL). Right columns: GCamp6 signals (green) in the same view fields before and after stimulation of the receptive field of joint nociceptors with the indicated stimuli. Arrows point to joint nociceptors in *Mrgprb2^+/+^* mice with increased GCamp6 fluorescence when the ankle was pressed with forceps, and 5 minutes after C48/80 (1.5 μg, 10 μL) was injected into the ankle joint, but not after vehicle (Veh; PBS; 10 μL) was injected. By contrast, no increase in GCamp6 fluorescence in joint sensory neurons from *Mrgprb2^–/–^* mice was observed after C48/80 injection. Scale bar: 100 μm. (**C**) Quantitative analysis of Ca^2+^ responses to vehicle and C48/80 in joint sensory neurons of WT mice (Veh: *n* = 8 mice; C48/80: *n* = 7 mice) and *Mrgprb2^–/–^* mice (Veh: *n* = 6 mice; C48/80: *n* = 6 mice). ***P < 0.001 vs. Veh; ^###^*P* < 0.001 vs. *Mrgprb2^+/+^*; 2-way ANOVA (repeated measures) followed by Bonferroni’s correction. (**D**) Size frequency distribution of joint sensory neurons of WT mice responding to vehicle (PBS; *n* = 8 mice) and C48/80 (*n* = 7 mice). ***P < 0.001 vs. Veh; unpaired 2-tailed Student’s *t* test.

To visualize whether synovial MC activation via Mrgprb2 directly activates peripheral terminals of joint nociceptors, we carried out in vivo calcium imaging on cell bodies of joint-innervating DRG neurons of *Pirt-Cre-GCamp6* mice, which express the fluorescent Ca^2+^ indicator GCAMP6 in primary sensory neurons (Figure 7A) (36). Injection of C48/80 but not vehicle (PBS) into the right hind ankle joint induced Ca^2+^ responses in a subpopulation of DiI-labeled joint sensory neurons with 5 minutes (Figure 7, B and C). The majority of C48/80-responding neurons were of small diameter (Figure 7D). Furthermore, neurons responding to C48/80 were also responsive to mechanical stimulation when the ankle was pressed with blunt forceps (Figure 7, B and C). However, the percentage of C48/80-responsive neurons was decreased in *Pirt-Cre-GCamp6* mice crossed onto *Mrgprb2^–/–^* mice (Figure 7, B and C). These data indicate that activation of synovial MCs via Mrgprb2 is able to activate peripheral terminals of joint sensory neurons in vivo.

### Neuropeptide SP serves as a key endogenous ligand to promote Mrgprb2/MRGPRX2 activation in GA.

After establishing that Mrgprb2 and MRGPRX2 are critical MC receptors activated in the context of GA, we determined which ligand(s) could regulate their activity. One potential candidate was SP, a common neuropeptide released from primary sensory fibers upon noxious stimuli and a known activator of Mrgprb2/MRGPRX2 (15, 16). The elevated SP level was observed in synovial fluid and synovial tissues in patients with GA (37).

**Figure 8:**
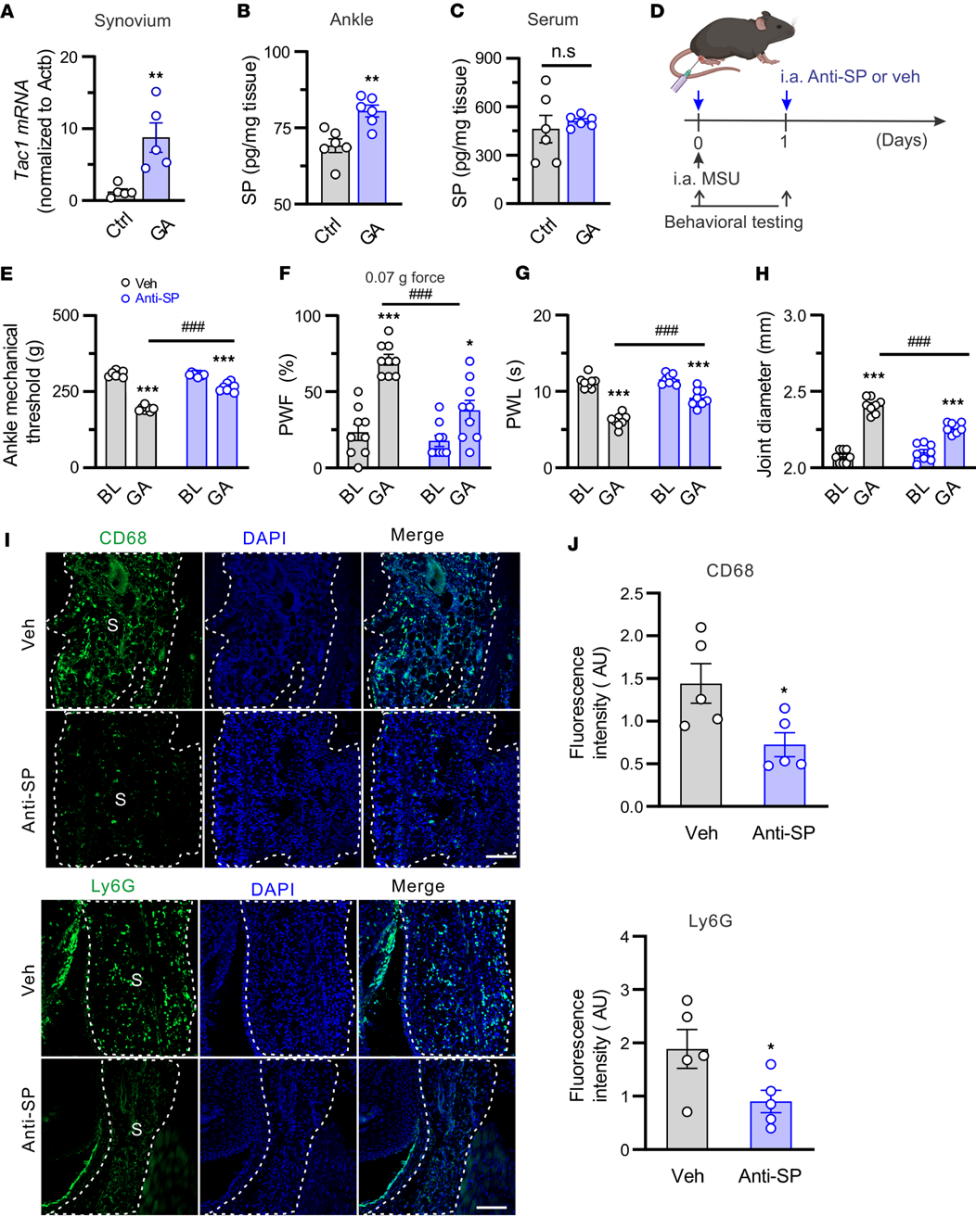
Neutralizing SP attenuates joint pain and inflammation in gout model mice. (**A**) Quantitative RT-PCR tests of *Tac1* mRNA expression in DRGs of control (Ctrl) and GA model mice. *n* = 5 mice per group; **P < 0.01 vs. Ctrl; unpaired 2-tailed Student’s *t* test. (**B** and **C**) SP ELISA test in ankle joint tissue (**B**) and serum (**C**) of control (Ctrl) and GA model mice. *n* = 6 mice per group; **P < 0.01 vs. Ctrl; unpaired 2-tailed Student’s *t* test. (**D**) Experimental schematic indicating the knee (for histology) or ankle joint (for behavioral testing) injected with anti-SP (15 μg, 10 μL) or vehicle (Veh; isotype control IgG; 15 μg, 10 μL). Figure created in BioRender (Qu L, 2026, https://BioRender.com/fyi3u5l). (**E**–**H**) Effects of SP-neutralizing antibody (*n* = 8 mice) or isotype control (Veh; *n* = 7 mice) on mechanical threshold in the ankle (**E**), paw withdrawal frequency (PWF) (**F**), paw withdraw latency (PWL) (**G**), and ankle diameter (**H**) in GA model mice. *P < 0.05, ***P < 0.001 vs. baseline (BL); ^###^*P* < 0.001 vs. Veh; 2-way ANOVA (repeated measures) with Bonferroni’s post hoc test. (**I**) Representative knee joint sections stained for CD68 or Ly6G. S, synovium. Scale bars: 100 μm. (**J**) Quantification showed that local neutralization of SP significantly reduced fluorescence intensity of Ly6G and CD68 staining in the synovium of GA mice compared with isotype control. *n* = 5 mice per group; *P < 0.05 vs. Veh; unpaired 2-tailed Student’s *t* test.

We performed quantitative PCR to assay for alterations in *Tac1* mRNA expression in mouse synovium on day 1 after GA when mice developed severe pain and inflammation. GA caused an upregulation of *Tac1* mRNA expression in mouse synovium 1 day after GA (Figure 8A). Also, ELISA revealed that SP protein levels were elevated in the synovium but not in the serum of gout model mice (Figure 8, B and C), indicating that SP is released locally. We next explored whether SP contributes to GA pain and joint inflammation by neutralizing SP in the joint cavity. To address this possibility, SP-neutralizing antibody or isotype control was injected into the ankle joints of WT mice once daily before and 1 day after GA (Figure 8D). Local administration of anti-SP antibody but not isotype control significantly alleviated primary mechanical hyperalgesia in the ankle, both secondary mechanical and thermal hyperalgesia in the hind ipsilateral hind paw, and joint swelling within 2 hours (Figure 8, E–H). Joint IHC analysis showed that neutralizing peripheral SP significantly decreased the infiltration of neutrophils (Ly6G) and macrophages (CD68) into the synovium of GA model mice compared with isotype control (Figure 8, I and J). These data indicate that local neutralization of SP in the joint of GA model mice alleviates pain hypersensitivity likely through a mechanism that depends on inflammation.

**Figure 9:**
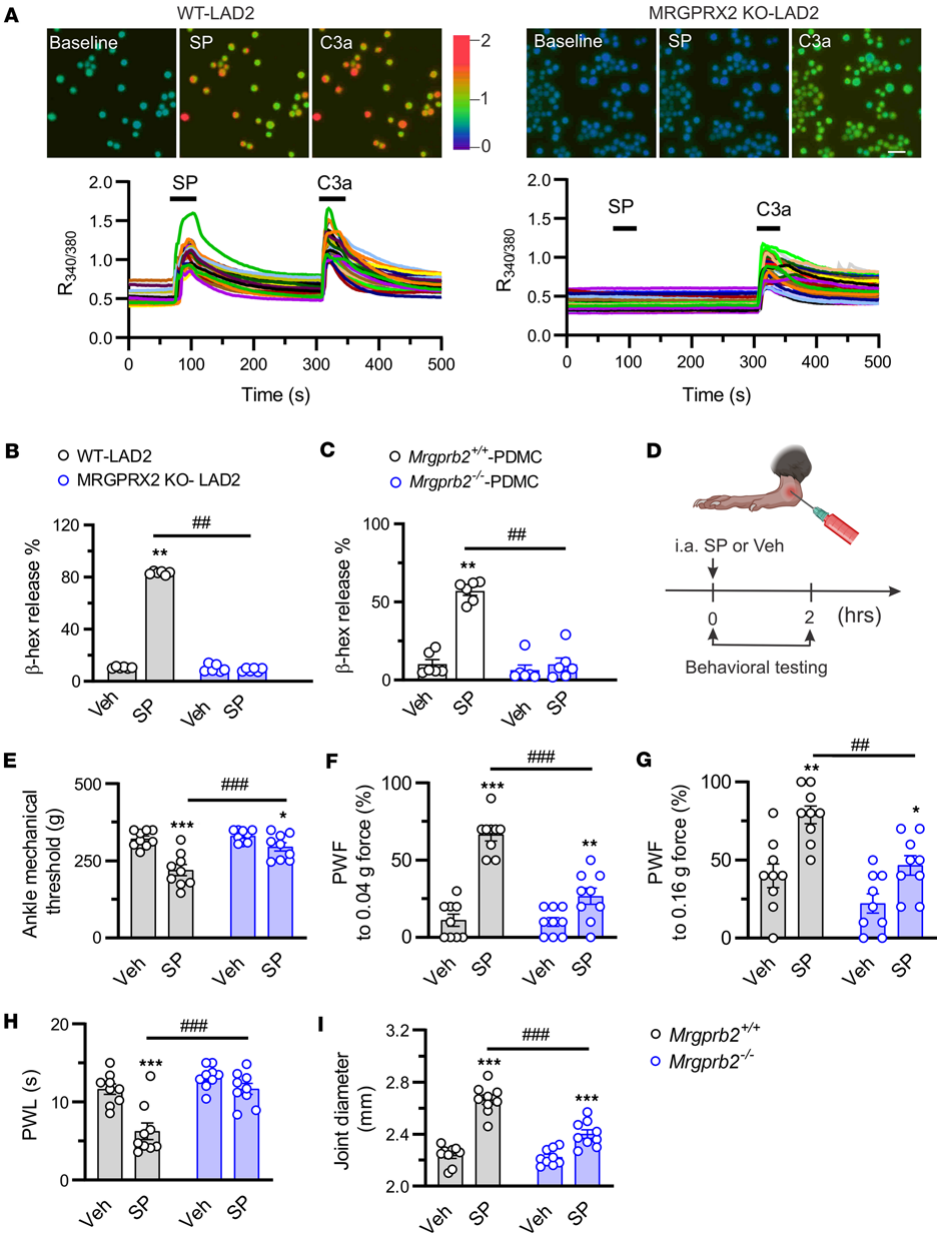
Activation of synovial Mrgprb2/MRGPRX2 by SP elicits pain hypersensitivity in naive mice. (**A**) Representative Ca^2+^ imaging (top) and time-lapse traces of changes in R_340/380_ (bottom) evoked by SP (1 μM) and C3a (50 nM) in WT (left) and MRGPRX2-KO (right) LAD2 cells (MRGPRX2-KO-LAD2). Scale bar: 50 μm. (**B**) β-Hexosaminidase (β-hex) release in WT and MRGPRX2-KO-LAD2 cells induced by SP (3 μM) and vehicle (Veh; PBS). *n* = 6 experiments per group; **P < 0.01 vs. Veh; ^##^*P* < 0.01 vs. WT-LAD2; 2-way ANOVA followed by Bonferroni’s correction. (**C**) β-Hexosaminidase (β-hex) release in PDMCs from *Mrgprb2^+/+^* (*n* = 6) and *Mrgprb2^–/–^* (*n* = 6) mice elicited by SP (100 μM) and vehicle (Veh; PBS). **P < 0.01 vs. Veh; ^##^*P* < 0.01 vs. *Mrgprb2^+/+^*; 2-way repeated-measures ANOVA with Bonferroni’s correction. (**D**) Intra-articular administration protocol of SP (100 μM, 10 μL) or vehicle (Veh; PBS; 10 μL). Behavioral measurements were performed within 2 hours after injection. Figure created in BioRender (Qu L, 2026, https://BioRender.com/snnmb6q). (**E**–**I**) *Mrgprb2^–/–^* mice exhibited higher mechanical threshold in the ankle (**E**), lower paw withdrawal frequency (PWF) responding to 0.04*g* (**F**) and 0.16*g* force (**G**), prolonged paw withdrawal latency (PWL) to radiant heat (**H**), and less joint swelling (**I**) following i.a. injection of SP compared with *Mrgprb2^+/+^* control littermates. *n* = 9 mice per group; *P < 0.05, **P < 0.01, ***P < 0.001 vs. Veh; ^##^*P* < 0.01, ^###^*P* < 0.001 vs. *Mrgprb2^+/+^* controls; 2-way ANOVA (repeated measures) followed by Bonferroni’s post hoc test.

Next, we sought to explore the cellular mechanisms whereby SP per se contributes to joint pain and inflammation. To assess whether SP activates human MCs via MRGPRX2, we performed Ca^2+^ imaging on WT LAD2 and MRGPRX2-KO-LAD2 cells. SP evoked robust Ca^2+^ responses in WT LAD2 cells but not MRGPRX2-KO-LAD2 cells (Figure 9A). However, C3a-induced Ca^2+^ responses were not impaired in MRGPRX2-KO-LAD2 cells (Figure 9A). In addition, SP triggered the release of β-hexosaminidase from WT LAD2 cells but not MRGPRX2-KO-LAD2 cells (Figure 9B). Similarly, SP failed to induce degranulation of PDMCs from *Mrgprb2^–/–^* mice compared with WT controls (Figure 9C). Considering MSU deposition as a major cause of GA, we tested whether MSU can activate MCs directly. Unfortunately, MSU had no significant effects on degranulation of WT LAD2 cells (Supplemental Figure 4). Next, we asked whether SP drives articular hypernociception via Mrgprb2 in vivo (Figure 9D). Injection of SP but not vehicle into right ankle joints of naive mice caused primary mechanical hyperalgesia in the ankle, secondary mechanical and thermal hyperalgesia in the hind paw, and joint swelling (Figure 9, E–I). However, such effects were diminished in *Mrgprb2^–/–^* littermates.

**Figure 10:**
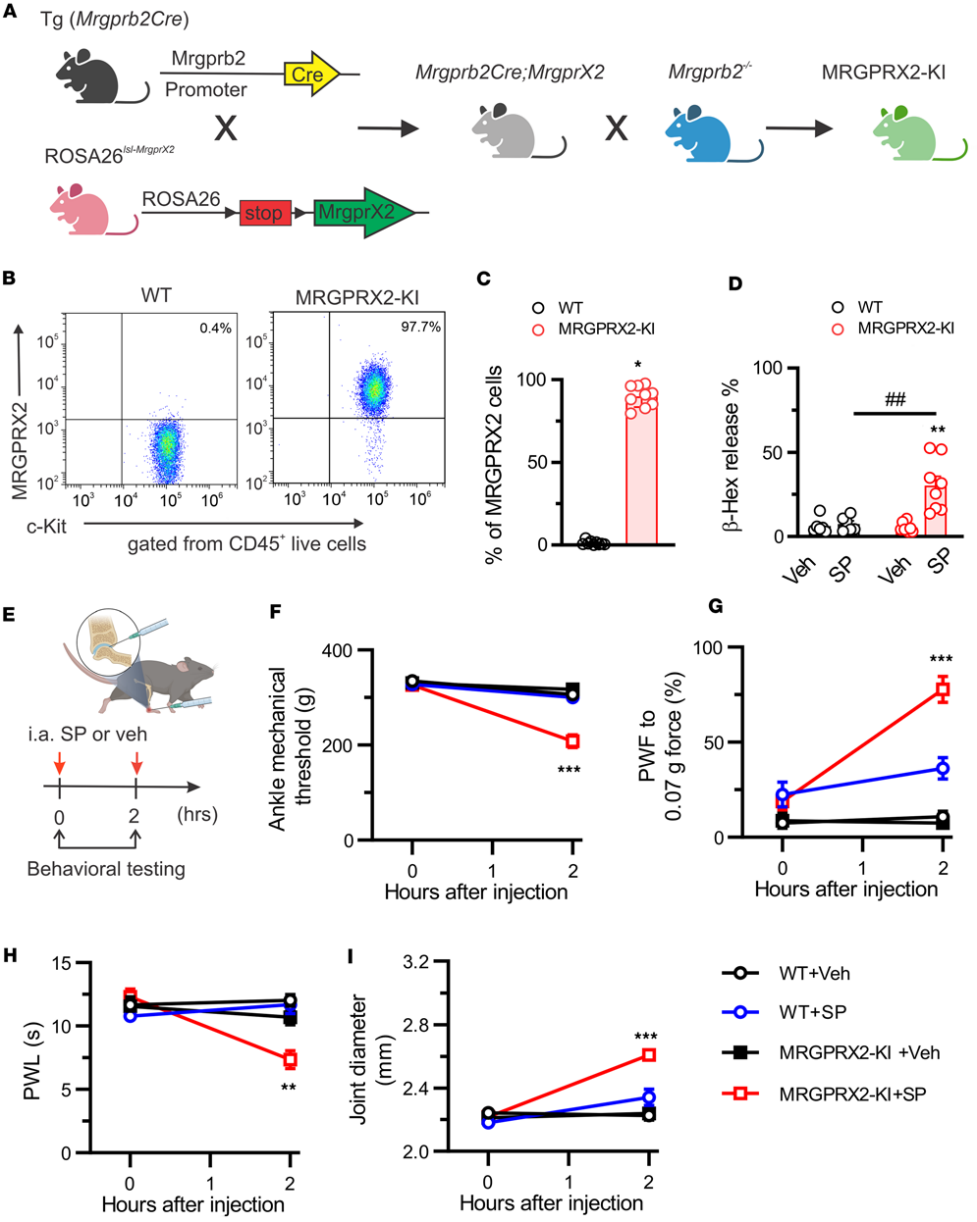
SP triggers behavioral signs of joint pain through human MRGPRX2 in humanized mice. (**A**) Generation of the MRGPRX2-KI transgenic mouse line. Figure created in BioRender (Qu L, 2026, https://BioRender.com/2xwxwpq). (**B**) Representative flow cytometric profile of *Mrgprb2Cre^–^* (WT) and MRGPRX2-KI PDMCs. PDMCs were identified as live CD45^+^c-Kit^+^ cells. (**C**) Flow cytometry assay confirmed the expression of MRGPRX2 in PDMCs isolated from MRGPRX2-KI mice but not *Mrgprb2Cre^–^* (WT) littermates. *n* = 9 mice per group; *P < 0.05 vs. WT; unpaired 2-tailed Student’s *t* test. (**D**) β-Hexosaminidase (β-hex) release in PDMCs from *Mrgprb2Cre^–^* (WT) and MRGPRX2-KI mice induced by SP (20 μM) and vehicle (Veh; PBS). *n* = 6–8 mice per group; **P < 0.01 vs. Veh; ^##^*P* < 0.01 vs. WT; 2-way ANOVA (repeated measures) followed by Bonferroni’s correction. (**E**) Experimental schematic showing i.a. administration protocol of SP (100 μM, 10 μL) or vehicle (Veh; PBS; 10 μL) as well as the time for behavioral testing after injection. Figure created in BioRender (Qu L, 2026, https://BioRender.com/rs679e9). (**F**–**I**) Comparisons of mechanical threshold in the ankle (**F**), paw withdrawal frequency (PWF) responding to 0.07*g* force (**G**), paw withdrawal latency (PWL) to radiant heat in the hind paw (**H**), and joint diameter (**I**) following i.a. injection of SP or vehicle between *Mrgprb2Cre^–^* (WT) and MRGPRX2-KI mice. *n* = 8–9 mice per group; **P < 0.01, ***P < 0.001 vs. WT; 2-way ANOVA (repeated measures) followed by Bonferroni’s correction.

To further translate the above mouse results to humans, we confirmed the action of SP in humanized mice that express human MRGPRX2 in mouse MCs lacking Mrgprb2 (MRGPRX2-KI). This mouse line was generated using the CRISPR/Cas9 editing approach as described previously (Figure 10A) (18). Flow cytometry assay confirmed the expression of MRGPRX2 in PDMCs of *Mrgprb2Cre*–positive mice but not *Mrgprb2Cre^–^* controls (Figure 10, B and C). Furthermore, SP induced degranulation of cultured PDMCs in *Mrgprb2Cre*–positive mice but not *Mrgprb2Cre^–^* mice, indicating that MRGPRX2 expressed on MCs is functional in humanized mice (Figure 10D). In addition, injection of SP, but not vehicle, evoked primary mechanical hyperalgesia in the ankle, secondary mechanical and thermal hyperalgesia in the hind paw, and joint swelling in MRGPRX2-KI mice (Figure 10, E–I). However, no such effects were observed in WT littermates (Figure 10, E–I). Thus, these translational data suggest that SP directly acts on the human ortholog MRGPRX2 to trigger joint pain and inflammation via the activation of synovial MCs.

## Discussion

Although MCs have been implicated in the pathogenesis of GA (6, 38, 39), the primary trigger for the activation of synovial MCs in GA still remains poorly understood. Previous studies have demonstrated that Mrgprb2/MRGPRX2 acts as a novel connective tissue MC (CTMC) receptor and is critical to the regulation of inflammatory responses and neurological outcomes (9, 15, 40). Yet its significance in GA pain and joint inflammation remains unknown. Here, we provide evidence that the MC-specific receptor Mrgprb2/MRGPRX2 acts as a key player in the development of arthritis pain and joint inflammation in the context of GA. Using transgenic reporter mice, we firstly detected that Mrgprb2-expressing MCs are present in the synovium of mice and are activated following GA. We also demonstrated that Mrgprb2 activation of synovial MCs is sufficient to activate joint-innervating sensory neurons, a conclusion supported by our in vivo DRG imaging showing that C48/80 can activate peripheral terminals of joint sensory neurons via a mechanism that depends on Mrgprb2 expressed on synovial MCs.

A second advance achieved by the present study is the revelation of a significant role for Mrgprb2 in modulating joint pain and inflammation in both naive and arthritis states. Chemical and/or chemogenetic activation of synovial Mrgprb2 triggered a similar magnitude of joint inflammation and behavioral signs of joint hypernociception in naive mice versus GA model mice. Genetic ablation of Mrgprb2-expressing MCs significantly attenuated acute joint pain hypersensitivity and inflammation induced by C48/80. In addition, acute pharmacological inhibition and global deletion of *Mrgprb2* each effectively alleviated arthritis pain and joint inflammation in GA as well as CFA arthritis models. Joint inflammatory process represents a key mechanism for GA pain (41, 42). In this study, two orthogonal and complementary lines of evidence support the notion that MC-expressed Mrgprb2 is necessary and sufficient to mediate joint hypernociception via a mechanism that is, at least in part, dependent on joint inflammation.

First, deleting *Mrgprb2* alleviated behavioral signs of joint pain in the GA model. These inhibitory effects are likely attributable to reductions in joint inflammation for the following reasons. Mrgrpb2 activity is known to mediate neutrophil infiltration in various inflammatory injuries (15, 40, 43). Our study extends previous findings by showing that deletion of *Mrgprb2* reduced neutrophil infiltration into the synovium during GA using flow cytometry assay and IHC staining. Considering that neutrophil influx into the synovium is a prominent feature of GA (44, 45), we suggest that Mrgprb2 contributes to the development of GA through the regulation of neutrophil infiltration in the inflamed joints. TNF-α, CXCL1, and IL-1β serve as critical regulators of neutrophil recruitment released by MCs (46–48). We observed that those genes were upregulated in the inflamed synovium following GA. Moreover, deletion of *Mrgprb2* decreased their upregulation. Therefore, these data suggest that Mrgprb2 signaling might drive the recruitment of neutrophils into the synovium via the regulation of TNF-α, CXCL1, and IL-1β release from MCs in the setting of GA. Future investigations will be necessary to assess this possibility. Although MC-deficient Kit^W-sh^/Kit^W-sh^ mice exhibit deficits in the development of neutrophils (i.e., neutrophilia) (49), we and other groups revealed that naive *Mrgprb2^–/–^* mice presented no such deficiencies in immunity (15). Thus, this reduction in immune cell infiltration observed in *Mrgprb2^–/–^* mice during GA is unlikely to be attributable to confounding immune abnormalities caused by *Mrgprb2* deletion.

Second, acute blockade of Mrgprb2 markedly reversed arthritis pain and joint swelling during GA. Joint IHC staining further confirmed that acute pharmacological inhibition of Mrgprb2 significantly reduced immune cell infiltration into the inflamed synovium. It is therefore likely that the analgesic effect of the Mrgprb2 inhibitor is secondary to the decrease of joint inflammation. Taken together, these findings point to Mrgprb2 as a critical regulator of immune cell recruitment in the context of GA. Given that the Mrgprb2 inhibitor was given systemically and global *Mrgprb2*-knockout mice were used in the present study, we cannot exclude the potential involvement of Mrgprb2-expressing MCs resident in other tissues besides the synovium.

Sensitization of joint nociceptors likely represents a key mechanism for GA pain (50, 51). As a reservoir for proinflammatory mediators, synovial MCs contribute to peripheral sensitization of joint sensory fibers via the modulation of chemokine and cytokine release (6). In this study, we found that global deletion of *Mrgprb2* decreased c-Fos expression in the superficial layer of dorsal horn of the spinal cord in GA model mice, suggesting that Mrgprb2 behaves as a potential key regulator of peripheral nociceptive inputs in the setting of GA. Furthermore, patch clamp recordings on isolated joint-innervating DRG neurons revealed that deleting *Mrgprb2* significantly reduced hyperexcitability of joint nociceptors following acute gout, indicating that Mrgprb2 is necessary to sustain aberrant peripheral nociceptive activity following GA. The exact molecular mechanisms whereby Mrgprb2 modulates the sensitization of joint nociceptors under GA conditions remain to be fully resolved. We found that Mrgprb2 is involved in the release of TNF-α, CXCL1, and IL-1β from MCs during GA. Those cytokines and chemokines are known to sensitize joint nociceptors to trigger joint pain hypersensitivity (52). Sphingosine 1-phosphate (S1P), a key bioactive molecule released by MCs, contributes to chronic pain and itch via the sensitization of peripheral nociceptors (53–55). We showed that Mrgprb2 regulated mRNA expression of sphingosine kinases (*Sphk1*) during GA, which is responsible for S1P synthesis. In addition, a recent study has demonstrated that MC-derived tryptase/PAR2 signaling contributes to the generation of chemotherapy-induced neuropathic pain via the modulation of peripheral sensitization (16). Further investigations will be necessary to address these possibilities in the setting of GA.

Bidirectional neuroimmune interactions are critical to the development of chronic pain and inflammation. Increasing evidence suggests that peripheral sensory neurons are of importance for the regulation of immunity through their release of neuropeptides (i.e., SP, VIP, CGRP) (56, 57). SP, a key neuropeptide, has been implicated in inflammation and chronic pain via regulation of immune cells (58). Recent studies have demonstrated that Mrgprb2/MRGPRX2 in skin MCs is required for SP-induced neurogenic inflammation, indicating that Mrgprb2/MRGPRX2 likely functions as a critical bridge between the nervous and immune systems (15). Our present study extends previous findings by introducing a neuroimmune mechanism for GA pain whereby SP acts as an endogenous agonist of Mrgprb2/MRGPRX2 that directly activates synovial MCs to promote GA pain. First, the mRNA and protein expression levels of SP were elevated in the synovium in the GA model. By contrast, no changes in SP protein expression were observed in the serum. Considering that SP^+^ sensory fibers are innervated in the synovium (59), we assume that SP release from sensitized joint sensory fibers might account for the elevated SP level in the synovium of GA mice. Second, neutralization of SP at the periphery, achieved with local injection of anti-SP antibody, significantly alleviated mechanical and thermal hyperalgesia and reduced joint swelling in the GA model. Joint IHC staining further confirmed that neutralization of SP reduced immune cell infiltration in the inflamed joints. These results indicate that SP directly promotes the development of inflammatory pain under GA conditions. Third, in naive mice, exogenous SP triggered nocifensive behaviors and joint swelling, whereas the pronociceptive effects of SP were diminished in mice lacking *Mrgprb2*. In vitro Ca^2+^ imaging and degranulation assay revealed that SP is able to activate MCs via Mrgprb2/MRGPRX2, consistent with previous reports (12, 15, 24). These findings suggest that SP directly modulates synovial MCs’ activity via Mrgprb2/MRGPRX2 to drive joint pain and inflammation. In addition to sensory neurons, SP can be secreted by a variety of immune cells (e.g., macrophages) (60). Thus, additional mechanisms besides neuroimmune interactions may contribute to SP–Mrgprb2/MRGPRX2–induced synovial MC activation. Such mechanisms will be studied in the future. Lastly, considering that species differences in the potency of SP for mouse Mrgprb2 and human MRGPRX2 may affect the translation of mouse studies to humans (12, 24), our studies used newly generated humanized mice that specifically express MRGPRX2 in MCs (18). We have demonstrated that local injection of exogenous SP is sufficient to evoke joint pain hypersensitivity and joint swelling via the regulation of primary MC degranulation through MRGPRX2 in vivo.

In summary, the present study illuminates a neuroimmune mechanism that might contribute to joint pain and inflammation in the setting of GA. We suggest that SP accumulated in the inflamed joints is sufficient to activate synovial MCs via Mrgprb2/MRGPRX2 to trigger GA-associated joint pain and inflammation. Our results reveal the MC receptor Mrgprb2/MRGPRX2 in the synovium as a key downstream effector of sensory neuron–MC interactions. Mrgprb2/MRGPRX2 activation of synovial MCs by the neuropeptide SP expands our understanding of the immunomodulation capability of primary sensory neurons. We suggest that MC-expressed Mrgprb2/MRGPRX2 merits consideration as a promising “druggable” target for GA management and other joint diseases involving MCs.

## Methods

Further information can be found in Supplemental Methods.

### Sex as a biological variable.

Our study examined male and female animals, and similar findings are reported for both sexes.

### Animals.

Animals were kept with a 14-hour light/10-hour dark cycle with ad libitum access to food and water. Adult mice (2–3 months) of both sexes on a C57BL/6 background were used in this study. The following mice were purchased from The Jackson Laboratory: Ai9 (stock 007909), Rosa26-DTA176 (stock 010527), and R26-LSL-Gq-DREADD (stock 026220). Adult C57BL/6J mice were obtained from The Jackson Laboratory (stock 000664) or Cyagen (C001089; Suzhou City, Jiangsu, China). Breeders of *Mrgprb2^–/–^* mice, *Mrgprb2Cre* mice, MRGPRX2-knockin mice, and *Pirt-Cre-GCamp6* mice were provided by Xinzhong Dong (Johns Hopkins University, Baltimore, Maryland, USA).

### MSU-induced GA model.

The GA model was induced by injection of MSU (MilliporeSigma) into a single joint of mice (42). Briefly, the mouse was under isoflurane (30% vol/vol) anesthesia, and the right hind ankle (for behavioral testing) or knee (for flow cytometry, joint histology, and PCR) was injected intra-articularly with 20 μL MSU (0.5 mg in sterile PBS) using a sterile insulin syringe with a 29-gauge needle. The sham control group received the same amount of vehicle (PBS) injections.

### Pain behavioral assessment.

Pain behavioral measurements were conducted on awake, free-moving, and age-matched littermates by an observer blinded to mouse genotype and treatment. Primary mechanical hyperalgesia in mouse ankle joints was evaluated by squeezing of the joint with a rodent pincher (Bioseb). A cutoff force of 350*g* was imposed to prevent joint damage. Mechanical threshold was defined as the force at which mice started to vocalize or withdraw their hind limbs forcefully (36, 61). A mean mechanical threshold in the joint was calculated by averaging of 3 repeated measurements obtained at the interval of at least 5 minutes. Secondary mechanical hyperalgesia in the hind paw was assessed using von Frey analysis. A series of filaments (0.04, 0.07, 0.16, 0.4, and 1.0*g*) were applied to glabrous skin of the hind paws. The number of paw withdrawal responses to 10 applications of each von Frey filament was counted as a percentage. Secondary thermal hyperalgesia was evaluated by radiant paw heating assay. Mice were acclimated to the testing environment for 2 hours daily for 2 days before measurements. Paw withdrawal latency to noxious heat stimuli was measured when a radiant heat source (IITC Life Science Inc.) was applied to the plantar area of the hind paw. A cutoff of 15 seconds was used to prevent tissue damage. Three repeated measurements of heat response latency were performed at least 3 minutes apart, and the average was used for analysis.

In some experiments, osthole (3 mg, 20 μL) or vehicle (1% DMSO in corn oil, 20 μL) was administered intraperitoneally (i.p.) once daily for 2 consecutive days. Pain behavioral assessments were conducted 1 hour after each injection.

### Immunohistochemistry.

Mice were anesthetized with urethane (1.5 g/kg, i.p.) and were perfused transcardially with PBS, followed by 4% paraformaldehyde (MilliporeSigma). Knee joint was dissected, decalcified in 10% EDTA for 2 weeks, and cryosectioned at 16 μm. After blocking with normal donkey serum (10%) for 1 hour at room temperature, sections were incubated with primary antibodies (Supplemental Table 1) overnight at 4°C. Sections were washed 3 times for 10 minutes and then incubated with the corresponding secondary antibodies (Supplemental Table 1) for 2 hours at room temperature. Images were captured using an upright confocal microscope (Nikon A1+ or Zeiss LSM 900).

### Fluorescent image analysis.

All images were analyzed by a blinded investigator using NIS Elements (Nikon) or ImageJ software (NIH). For joint tissues, 2–3 sections per animal were imaged and analyzed. The number of cells immunopositive for a given marker (CD45, Ly6G, CD68, mCherry, and c-Kit) per unit area or the fluorescence intensity per unit area was used for signal quantification. For a given marker, all parameters used for image acquisition and analysis were kept consistent across animals.

### Intra-articular injection of C48/80, CNO, SP, and anti-SP antibody.

Compound 48/80 (C48/80; 1.5 μg, 20 μL; MilliporeSigma), clozapine *N*-oxide (CNO; 20 μg, 10 μL; Tocris), or SP (100 μM, 10 μL; Tocris) was injected into a single ankle joint of naive mice. Control animals received the same amounts of PBS. The concentration used was chosen based on previous reports and our pilot studies. For some experiments, either anti-SP antibody (15 μg in 10 μL saline; Supplemental Table 1) or isotype control (normal rabbit serum; Supplemental Table 1) was injected into the right knee or ankle of mice on days 0 and 1 after MSU challenge. Pain-related behaviors, joint diameter, and immunostaining were measured over the ensuing 2 hours after injection.

### Retrograde labeling of joint-innervating DRG neurons.

To retrogradely label joint-innervating DRG neurons, 1,1′-dioctadecyl-3,3,3′,3′-tetramethylindocarbocyanine perchlorate (DiI; MilliporeSigma) was injected into the right ankle (2.5 mg/mL, 8 μL in 25% ethanol) of mice at least 7 days before experiments (36). DRG neurons that innervated the joint were identified by the presence of DiI in cell bodies.

### In vivo DRG calcium imaging.

Ca^2+^ imaging was conducted on the DRG of WT and *Mrgprb2^–/–^* mice on *Pirt-Cre-GCamp6* background. On day 7 after DiI injection, mice were anesthetized with 1.5% isoflurane delivered via nose cone. After the lumbar vertebral column was exposed, a dorsal laminectomy was conducted at L1–L6 levels. The L4 DRG was then exposed and submerged in oxygenated artificial cerebrospinal fluid (ACSF) within a pool formed by a ring to which the back skin was sewn. The ACSF solution contained 130 mM NaCl, 3.5 mM KCl, 1.2 mM MgCl_2_, 1.25 mM NaH_2_PO_4_, 24 mM NaHCO_3_, 1.2 mM CaCl_2_, and 10 mM dextrose (pH 7.4, bubbled with 95% O_2_ and 5% CO_2_; 290–310 mOsm). Two pairs of custom-designed clamps were used to stabilize mouse vertebrae. All DRG images were acquired using a Nikon A1+ confocal microscope as described in our previous study (36). For GCamp6 excitation, a laser wavelength at 488 nm was used, and GCamp6 green fluorescence signals were recorded at the wavelength of 500–550 nm. The entire DRG image was captured using a ×10 water immersion lens at the resolution of 512 × 512 pixels using *Z*-stacks in 7–11 steps over a distance of 175–300 μm. For a given stimulus, each stack took 20–30 seconds to achieve, and a total of 20–35 *Z*-stacks per DRG were taken. To circumvent a potential priming effect of different chemicals, either C48/80 (1.5 μg, 10 μL) or vehicle (saline, 10 μL) was injected into a single ankle joint cavity. Noxious mechanical stimulation was applied to the ankle joint using serrated forceps at the end**)** of each experiment. The interval between stimuli was set at 3–5 minutes. All in vivo images were analyzed using ImageJ software by investigators blinded to groups. Motion artifacts in images were corrected using Linear Stack Alignment plug-ins in ImageJ. All retrogradely DiI-labeled neurons with increased fluorescence upon stimulation were chosen as regions of interest for further analysis. Neurons were ruled out if they exhibited spontaneous Ca^2+^ responses in the absence of stimulation. A neuron was considered responsive to a given stimulus if Δf/F_0_ was greater than 15%, where F_0_ refers to the baseline of fluorescence intensity before stimulation (36).

### DRG neuron culture.

Mouse DRG neurons were acutely dissociated and cultured as reported previously (30, 62). One week after DiI labeling, L3–L5 lumbar DRGs were harvested and placed in ice-cold complete saline solution (CSS) containing (in mM): 137 NaCl, 5.3 KCl, 25 sorbitol, 3 CaCl_2_, 1 MgCl_2_, and 10 HEPES (pH 7.2 with NaOH). After careful cleaning and trimming, DRGs were digested in an enzyme solution with Liberase TM (0.35 U/mL; Roche Diagnostics Corp.) and EDTA (0.5 mM) in CSS for 20 minutes at 37°C, and then digested for 15 minutes with Liberase TL (0.25 U/mL; Roche Diagnostics Corp.) supplemented with papain (20–30 U/mL; Worthington Biochemical) and 0.5 mM EDTA in CSS at 37°C. After digestion and centrifugation, the tissue was resuspended in DMEM (Life Technologies Corp.) plus 10% fetal bovine serum (FBS; MilliporeSigma) and 1% penicillin and streptomycin (Invitrogen). After mechanical trituration with a fire-polished Pasteur pipette, neurons were spotted onto poly-d-lysine– and laminin-coated coverslips for 45 minutes at 37°C and then flooded with the complete DRG medium described above. DRG neurons were maintained in a humidified incubator in 5% CO_2_ at 37°C, and used for patch clamp experiments within**)** 16–24 hours.

### Whole-cell recordings in DRG neurons.

Whole-cell recordings were carried out on DiI-labeled small-diameter (≤25 μm) joint-innervating DRG neurons with a MultiClamp700B amplifier and Digidata1550B digitizer (Molecular Devices) (30). Patch pipettes with a resistance of 4–5 MΩ were fabricated from borosilicate glass capillaries (Sutter Instruments) using a horizontal pipette puller P97 (Sutter Instruments). Electrophysiological signals were low-pass-filtered at 2 kHz and sampled at 10 kHz using pClamp11 software (Molecular Devices). Patch pipettes were filled with an internal solution containing 120 mM K^+^-gluconate, 1 mM CaCl_2_, 20 mM KCl, 2 mM MgCl_2_, 11 mM EGTA, 10 mM HEPES-K^+^, 2 mM MgATP (pH 7.2 with Tris base; 290–300 mOsm). Neurons were bathed in an external solution containing 145 mM NaCl, 2 mM MgCl_2_, 3 mM KCl, 2 mM CaCl_2_, 10 mM HEPES, 10 mM glucose (pH 7.4 with NaOH). In current clamp mode, resting membrane potential (RMP) of recorded neurons was monitored within 3 minutes after stabilization. A neuron with RMP more negative than –40 mV and the spike overshoot greater than 15 mV was acceptable. Cell capacitance (pF) was read directly from the MultiClamp700B amplifier control panel. Liquid junction potential corrections (11 mV) were applied after recordings. A step protocol of depolarizing currents (20 pA steps from 0 pA to 300 pA for 350 milliseconds) was applied to evaluate the rheobase, RMP, and number of action potentials. Input resistance was assessed from the slope of a current-voltage plot responding to a series of hyperpolarizing current steps (50 pA steps from –200 to –50 pA for 350 milliseconds). Data were analyzed with Clampfit 11.0 software (Molecular Devices).

### Human mast cell line culture.

WT LAD2 (Laboratory of Allergic Diseases 2) human mast cells (MCs) (gift of Arnold Kirshenbaum and Dean Metcalfe, NIH, Bethesda, Maryland, USA) and MRGPRX2-KO-LAD2 cells (gift of Xinzhong Dong) were cultured in StemPro-34 SFM medium (Life Technologies) supplemented with 2 mM l-glutamine, 100 ng/mL recombinant human stem cell factor (PeproTech), and 1% antibiotics (penicillin and streptomycin). Cells were cultured at a density of 1 × 10^5^ cells/mL and maintained at 37°C and 5% CO_2_. Cell culture medium was hemi-depleted once per week with fresh medium. The purity of LAD2 cells was periodically tested for the expression of CD117 and FcεRI using flow cytometry. The loss of MRGPRX2 expression in LAD2 cells was validated by flow cytometry using anti–human MRGPRX2 and anti–human CD117 antibodies (Supplemental Table 1).

### Mouse peritoneal-derived mast cell culture.

Peritoneal-derived mast cells (PDMCs) of adult mice were cultured as described previously (18). The peritoneal cavity of WT, *Mrgprb2^–/–^*, and MRGPRX2-KI mice was lavaged with ice-cold MC dissociation medium (MCDM; Hanks balanced salt solution buffer with 3% FBS and 10 mM HEPES). Cells (1 × 10^5^ cells/mL) were resuspended and cultured in RPMI 1640 supplemented with 10% FBS, 30 ng/mL recombinant murine stem cell factor (mSCF; PeproTech), 10 ng/mL recombinant murine IL-3 (PeproTech), and 1% antibiotics (penicillin and streptomycin) for 2 days. On day 3, non-adherent cells were removed, and adherent cells were cultured in a fresh complete medium for an additional 6–7 days. PDMCs were used for experiments only if the purity was greater than 90% as tested by flow cytometry using anti-CD117 and anti-FcεRI antibodies (Supplemental Table 1).

### β-Hexosaminidase release assay.

Cultured mouse PDMCs (1 × 10^5^ cells) or LAD2 cells (2.5 × 10^4^ cells) were seeded into a 96-well plate with a total volume of 50 μL of BSA-HEPES buffer (same as the external solution used for patch clamp experiments) containing 0.4% BSA and treated with 50 μL of C48/80 (10 μg/mL for LAD2 cells, 50 μg/mL for PDMCs), SP (10 μM for LAD2 cells, 100 μM for PDMCs), C3a (300 nM for LAD2 cells), or CNO (10 μM for PDMCs) for 30 minutes at 37°C. Cells stimulated with vehicle (PBS) served as a control. After centrifugation at 200*g* for 5 minutes, the supernatant was transferred to a new flat-bottom 96-well plate. The remaining cell pellets were then lysed with 50 μL of 0.1% Triton X-100 in BSA-HEPES buffer and transferred to a new flat-bottom 96-well plate. Supernatants and cell lysates were then incubated with 50 μL of *p*-nitrophenyl *N*-acetyl-β-d-glucosamine (PNAG; MilliporeSigma) in 0.1 M sodium citrate buffer (pH 4.5) and incubated at 37°C for 90 minutes. Glycine buffer (0.4 M, 50 μL) was added to end the reaction. The absorbance was evaluated at 405 nm and 570 nm using a SpectraMax i3x microplate reader (Molecular Devices). β-Hexosaminidase release was calculated by the percentage of total release.

### Mouse synovial MC degranulation quantification.

Degranulated MCs were identified as cells that possessed at least 5 extracellular vesicles with a diameter of 0.8–1.5 μm (to rule out background signals/autofluorescence) within a distance of 5 μm from the cell surface as reported previously (40). The number of synovial MCs and degranulated MCs across all images was counted by investigators blinded to genotype and treatment.

### Statistics.

Statistical analysis was performed using GraphPad Prism 8.0 software. Data are presented as means ± SEM. A 2-tailed Student’s *t* test was performed for the comparison of 2 groups. A 1-way or 2-way ANOVA for random measures or repeated measures with Bonferroni’s post hoc test was used for comparisons of multiple groups or multiple time points. Statistical significance was defined as *P* < 0.05. Sample size and the type of statistical tests used for each comparison are indicated in the figure legends.

### Study approval.

All animal experimental procedures were approved by the Institutional Animal Care and Use Committee of the University of South China (ethical approval no. USC2024XS056) and were performed according to the guidelines of the NIH and the International Association for the Study of Pain.

### Data availability.

All data supporting the findings in the main article and the supplemental material are available in the Supporting Data Values file.

## Author contributions

LY performed behavioral tests, quantitative PCR, and ELISA and analyzed the data. CL designed the experiments, assisted with data analysis, and revised the manuscript. JX and YS performed IHC staining and data analysis. DL assisted with quantitative PCR and mouse genotyping. HC, CZ, DQ, NL, TH, YL, and WL facilitated experimental design and data analysis and revised the manuscript. LQ conceived the entire project, designed the experiments, conducted behavioral tests and in vivo and in vitro calcium imaging, analyzed the data, and wrote the manuscript.

## Funding support

Natural Science Foundation of China (82471235 to LQ; 82301447 to CL).

- Key Project of Hunan Province Natural Science Funds (2025JJ90129 to LQ).
- Key R&D Program of Hunan Province Natural Science Funds (2024JK2131 to WL).
- Hunan Clinical Research Center for Acute and Chronic Pain (2023SK4014 to WL).
- Hunan Clinical Research Center for Obesity Related Diseases (2023SK4052 to YL).
- Research startup funds of the Second Affiliated Hospital at University of South China (2023G02 to LQ).

## Supplementary Material

Supplemental data

Supporting data values

## Version 1 01/23/2026

Electronic publication
