# Supplementary material for: Mast cell–expressed Mrgprb2/MRGPRX2 mediates gout pain and inflammation via a neuroimmune axis
Source: JCI Insight. 2026 Jan 23;11(2):e201781. doi: 10.1172/jci.insight.201781 (PMC12892919; doi:10.1172/jci.insight.201781)
Supplement: Supplemental data [file jciinsight-11-201781-s224.pdf]

Supplementary material

Supplementary methods

Complete Freund adjuvant (CFA)-induced inflammatory arthritis model

Inflammatory arthritis model was established through i.a. injection of CFA (1 mg/ml; 5 µl; Sigma, St Louis, MO) into right hind ankle joint of mice under isoflurane (30% vol/vol) anesthesia. Nocifensive behaviors and joint swelling were evaluated over a 2- week period.

Flow cytometry

On day 1 after gout induction, synovial tissues were collected from mouse knees and placed in cold RPMI 1640 medium containing 0.15 mg/mL Hyalauronidase (Sigma, St Louis, MO), 1 mg/mL Collagenase type IA (Sigma, St Louis, MO), and 1 mg/mL Collagenase VIII (Sigma, St Louis, MO) followed by incubation at 37°C for 1 hour. After digestion, synovial tissues were filtered with a 70 µm cell strainer, centrifuged and washed with 0.5% BSA in PBS. For LAD2 cells or PDMCs, cells were centrifuged at 300 g for 5 min and resuspended in 0.5% BSA in PBS. Cells were incubated with CD16/CD32 Fc block (1:100; eBioscience, Santa Clara, CA) for 10 min before the addition of specific antibodies. All antibodies used in this experiment were listed in Table S1. Non-viable cells were excluded by staining with 7-Aminoactinomycin D (7-AAD; 1:100; BD Pharmingen, San Diego, CA). Samples were captured with a CytoFLEX LX flow cytometer (Beckman Coulter, Indianapolis, IN). Data analysis was performed using CytExpert 2.3 (Beckman Coulter, Indianapolis, IN) or FlowJo™ 10 software (TreeStar, Ashland, OR).

Quantitative real-time PCR (qRT-PCR)

On day 1 after GA induction, mouse synovium from different groups was freshly harvested. Total RNA was then extracted using a RNeasy lipid tissue mini kit (Qiagen). A NanoDrop 1000

(Thermo Fisher Scientific, Waltham, MA) was used to measure RNA quantity. Complimentary DNA (cDNA) was generated using the iScript cDNA synthesis kit (Vazyme, Nanjing, China) according to the manufacturer's manual. RT-PCR was performed on a QuantStudio 3 real-time PCR system or a QuantStudio 5 real-time PCR system (Applied Biosystems, Waltham, MA) using PowerUp SYBR Green Master Mix (Vazyme, Nanjing, China). The sequence of primers used was listed (Supplemental Table 2). Each sample was run in duplicate. The mRNA expression levels of target genes were analyzed using the $2^{-\Delta\Delta CT}$ method and was normalized to that of *Actb*.

In vitro calcium imaging assay

LAD2 cells (1×10^5) were loaded with 1 μM Fura-2-acetoxymethyl ester (Fura-2 AM; Invitrogen, Vilnius, Lithuania) in the loading buffer (same as the external solution used for patch clamp experiments) for 40-50 min at 37°C. Cells were subsequently washed three times and imaged in the external solution (same as those used for patch clamp experiments) at room temperature. An invert fluorescent microscope (Nikon ECLIPSE TE200; Melville, NY) was used for ratiometric Ca^{2+} imaging. Fluorescent images under 340 and 380 nm excitation wavelengths were captured at 2 s intervals using a cooled CCD camera (ANDOR, Belfast, Northern Ireland) driven by a NIS-Elements software. The ratio of fluorescence intensity [$R_{(340/380)}$] obtained at 340 nm to 380 nm within a certain region of interest was used to reflect the changes in intracellular Ca^{2+} signals. A cell was considered responsive to a stimulus if a change with R340/380 was equal or greater than 15% above baseline. SP (1 μM; Tocris, Bristol, UK) and complement 3a (C3a; 50 nM; Sigma, St Louis, MO) were applied to stimulate LAD2 cells.

ELISA assay

On day 1 after GA induction, synovial tissues were harvested from the knee of mice under urethane (1.5 g/kg; i.p.) anesthesia. The freshly diced tissues were homogenized with a high-speed KZ-III tissue homogenizer (Servicebio, Wuhan, China) and then centrifuged at 10,000 g for 10 min at 4°C. For mouse blood, serum was collected by centrifuging blood samples at 1,000 g for 10 min at 4°C. All the supernatants were harvested and tested for SP levels using a SP mouse ELISA kit (BBI Life Science, Shanghai, China) according to the manufacturer's instructions. The plate was read at 450 and 570 nm using a microplate reader (Molecular Devices, Sunnyvale, CA). The BCA assay kit (Beyotime, Shanghai, China) was used to quantify total protein of each sample.

Supplementary figure legends

Supplementary Figure 1. No sexual dimorphism in analgesic effects of *Mrgprb2* deletion was observed in GA model mice. (A-H) Comparison of mechanical threshold in the ankle (**A, E**), paw withdrawal frequency to 0.16 g in the hind paw (**B, F**), paw withdrawal latency (PWL) to radiant heat in the hind paw (**C, G**), joint diameter (**D, H**) between male (*Mrgprb2*$^{+/+}$, n = 10 mice; *Mrgprb2*$^{-/-}$; n = 9 mice) and female (*Mrgprb2*$^{+/+}$, n = 7 mice; *Mrgprb2*$^{-/-}$; n = 6 mice) mice of each genotype over the course of GA. *p< 0.05, **p < 0.01, ***p < 0.001 versus *Mrgprb2*$^{+/+}$; two-way ANOVA for repeated measures followed by Bonferroni post hoc test.

Supplementary Figure 2. Genetic deletion of *Mrgprb2* attenuates joint pain in CFA induced arthritis model. (**A-D**) Time course of ankle mechanical threshold (**A**), paw withdrawal frequency to 0.07 g (**B**), paw withdrawal latency to radiant heat (**C**), and ankle joint diameter (**D**) following intra-articular (i.a.) injection of CFA in *Mrgprb2*$^{+/+}$ (n = 8 mice) and *Mrgprb2*$^{-/-}$ mice (n = 7 mice); *p < 0.05, ** p < 0.01, ***P < 0.001 versus *Mrgprb2*$^{+/+}$, two-way repeated measures ANOVA followed by Bonferroni post hoc test.

Supplementary Figure 3. *Mrgprb2*$^{-/-}$ mice exhibit no deficits in the number of synovial MCs. (**A**) Representative sections of knee joints from *Mrgprb2*$^{+/+}$ and *Mrgprb2*$^{-/-}$ mice and stained with c-Kit and DAPI. S: synovium. Scale bar: 100 µm. (**B**) No significant difference in the number of synovial MCs was observed between genotypes. n = 5 mice per group; P > 0.05; unpaired Student's t test.

Supplementary Figure 4. MSU fails to induce degranulation in LAD2 cells. (**A**) β hexosaminidase (β-hex) release in LAD2 cells induced by MSU (250, 500, 1000 µg/ml), C48/80

(20 μg/ml) and vehicle (Veh; PBS). n = 8 experiments per group. ***p < 0.001 versus Veh; one-way ANOVA followed by Tukey's test.

Supplementary Figure

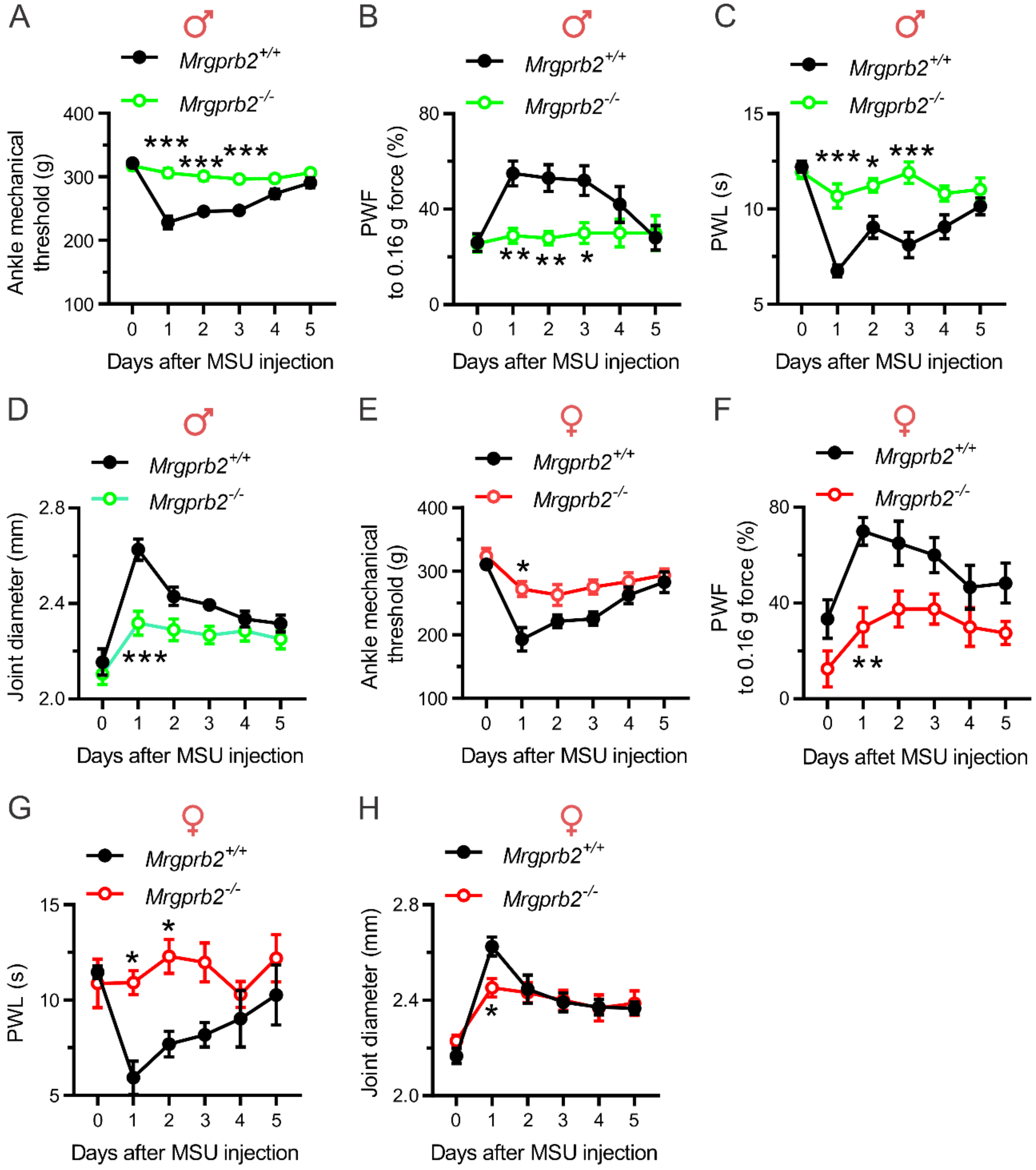

Supplementary Figure 1. No sexual dimorphism in analgesic effects of *Mrgprb2* deletion was observed in GA model mice. (A-H) Comparison of mechanical threshold in the ankle (**A, E**), paw withdrawal frequency to 0.16 g in the hind paw (**B, F**), paw withdrawal latency (PWL) to radiant heat in the hind paw (**C, G**), joint diameter (**D, H**) between male (*Mrgprb2*$^{+/+}$, n = 10 mice; *Mrgprb2*$^{-/-}$; n = 9 mice) and female (*Mrgprb2*$^{+/+}$, n = 7 mice; *Mrgprb2*$^{-/-}$; n = 6 mice) mice

of each genotype over the course of GA. *p< 0.05, **p < 0.01, ***p < 0.001 versus *Mrgprb2*$^{+/+}$; two-way ANOVA for repeated measures followed by Bonferroni's post hoc test.

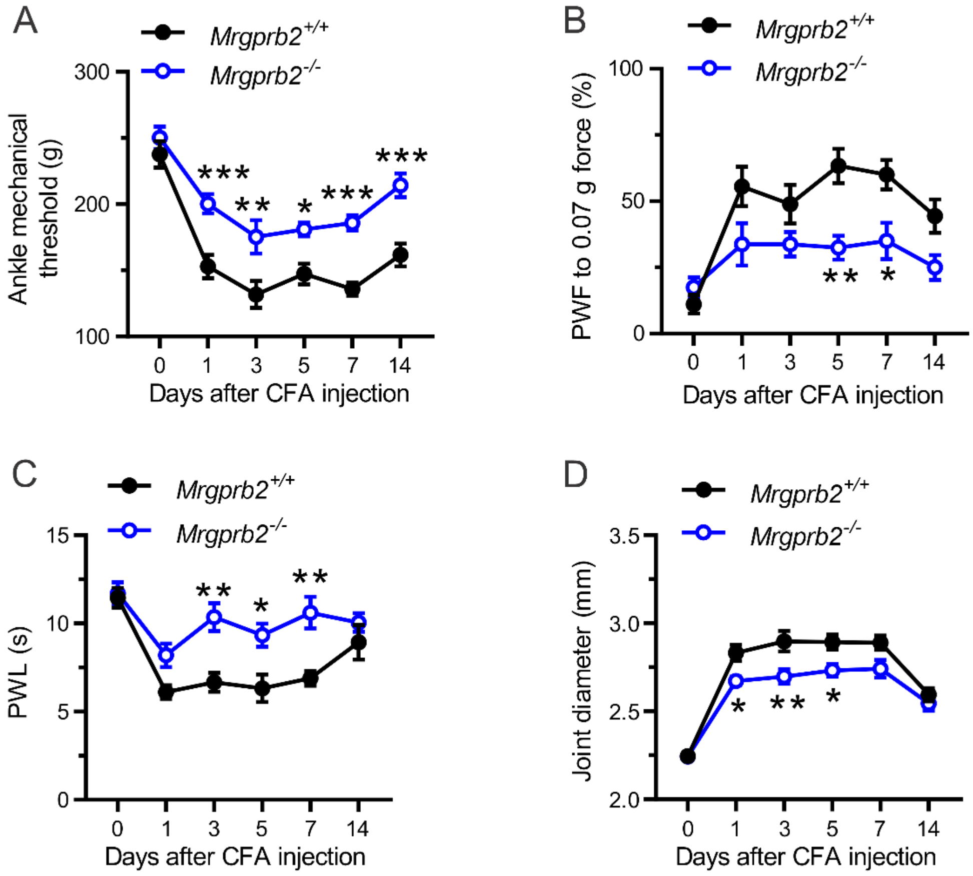

Supplementary Figure 2. Genetic deletion of *Mrgprb2* attenuates joint pain in CFA induced arthritis model. (**A-D**) Time course of ankle mechanical threshold (**A**), paw withdrawal frequency to 0.07 g (**B**), paw withdrawal latency to radiant heat (**C**), and ankle joint diameter (**D**) following intra-articular (i.a.) injection of CFA in *Mrgprb2*$^{+/+}$ (n = 8 mice) and *Mrgprb2*$^{-/-}$ mice (n = 7 mice); *p < 0.05, ** p < 0.01, ***P < 0.001 versus *Mrgprb2*$^{+/+}$, two-way repeated measures ANOVA followed by Bonferroni's post hoc test.

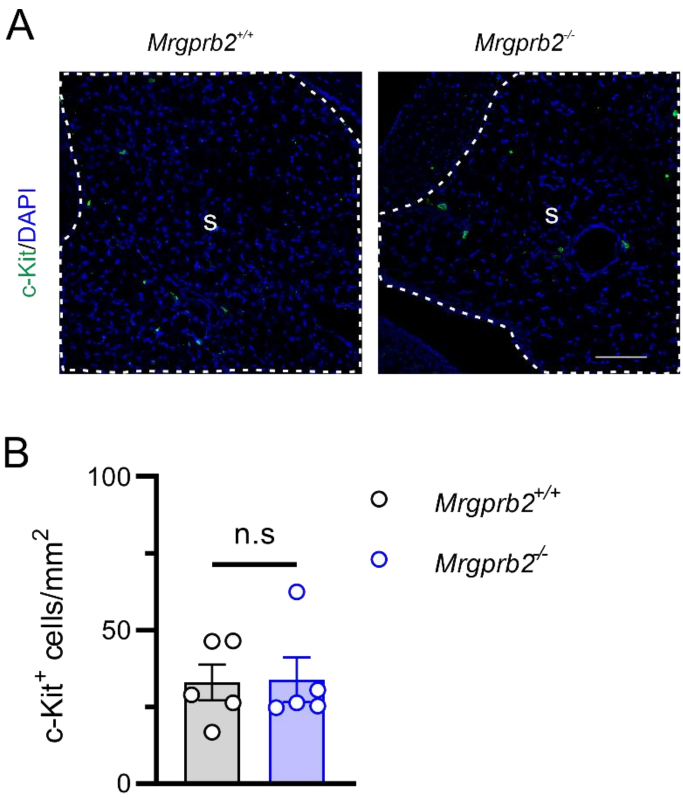

Supplementary Figure 3. ***Mrgprb2***$^{-/-}$ **mice exhibit no deficits in the number of synovial MCs.** (**A**) Representative sections of knee joints from *Mrgprb2*$^{+/+}$ and *Mrgprb2*$^{-/-}$ mice and stained with c-Kit and DAPI. S: synovium. Scale bar: 100 µm. (**B**) No significant difference in the number of synovial MCs was observed between genotypes. n = 5 mice per group; $P > 0.05$; unpaired Student's t test.

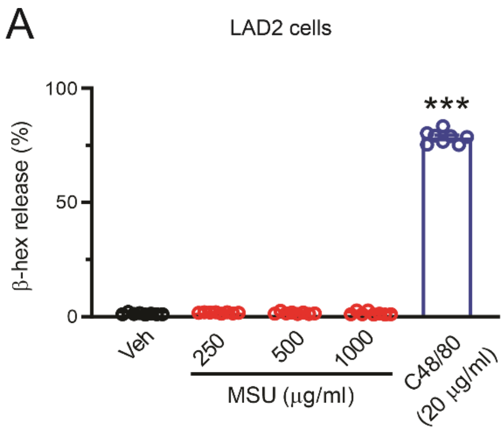

Supplementary Figure 4. MSU fails to induce degranulation in LAD2 cells. (**A**) β hexosaminidase (β-hex) release in LAD2 cells induced by MSU (250, 500, 1000 µg/ml), C48/80 (20 µg/ml) and vehicle (Veh; PBS). n = 8 experiments per group. ***p < 0.001 versus Veh; one-way ANOVA followed by Tukey's test.

Table S1. Key resources table

Regent or Resource	Dilution	Source	Identifier
Antibodies			
Goat anti-c-Kit	1:1000	R&D system	Cat# AF1356
Rat anti-CD45	1:500	Sigma	Cat# 05-1416
Rat anti-CD68	1:500	Biolegend	Cat# 137001
Rat anti-Ly6G	1:1000	Biolegend	Cat# 127601
Rabbit anti-PGP9.5	1:1000	Abcam	Cat# ab108986
Rabbit anti-HA	1:200	Cell Signaling Technology	Cat# 3724
Rabbit anti-c-Fos	1:1000	Sigma	Cat# ABE457
Chicken anti-NeuN	1:200	Aves	Cat# NUN-0020
Goat anti-mCherry	1:250	ORIGENE	Cat# AB0040
Donkey anti-Rat IgG Alexa 488	1:250	Jackson ImmunoResearch labs	Cat# 712-545-153
Donkey anti-Rabbit IgG Alexa 488	1:250	Jackson ImmunoResearch labs	Cat# 711-545-152
Donkey anti-Rabbit IgG Alexa 647	1:250	Jackson ImmunoResearch labs	Cat# 711-605-152
Donkey anti-Goat IgG Alexa 488	1:250	Jackson ImmunoResearch labs	Cat# 705-545-147
Donkey anti-Chicken Alexa 488	1:250	Jackson ImmunoResearch labs	Cat# 703-545-155
Donkey anti-Goat IgG Alexa 594	1:250	Abcam	Cat# ab150132
PE anti -mouse CD45	1:100	Biolegend	Cat# 103105
FITC anti-mouse CD11b	1:100	Biolegend	Cat# 101205
APC anti-mouse Ly6G	1:100	Biolegend	Cat# 127613
APC anti-mouse c-Kit	1:100	Biolegend	Cat# 135108
FITC anti-mouse FcεRI	1:100	Biolegend	Cat# 134305
PE anti-human CD117	1:100	Milltenyi Biotec	Cat# 130114070
APC anti-human MRGPRX2	1:100	Biolegend	Cat# 359005
FITC anti-human CD45	1:100	Biolegend	Cat# 304006
Anti-mouse CD32/16 Fc Blocker	1:100	eBioscience	Cat# 14016182
Chemicals, peptides, reagents			

Avidin-FITC	Sigma	Cat# A2050
MSU	Sigma	Cat# U2875
CFA	Sigma	Cat# F5881
7-AAD	BD Pharmingen	Cat# 516898
Substance P	Tocris	Cat# 1156
Compound 48/80	Sigma	Cat# C2313
Complement 3a	Sigma	Cat# 204881
CNO	Tocris	Cat# 4936
Osthole	MCE	Cat# HY-N0054
Dil	Sigma	Cat# 42364
Fura-2 AM	Invitrogen	Cat# F1221
PNAG	Sigma	Cat# 487052
Anti-SP antibody for pain studies	Millipore Sigma	Cat# AB1566
Isotype IgG for pain studies	Senbeijia	Cat# SBJ-SE-RAB0001
Liberase TM	Roche Diagnostics Corp	Cat# 0540119001
Liberase TL	Roche Diagnostics Corp	Cat# 05401020001
Papain	Worthington Biochemical	Cat# LS003126
Bovine serum albumin	Sigma	Cat# A9418
Hyaluronidase	Sigma	Cat# H3506
Collagenase type IA	Sigma	Cat# C9891
Collagenase VIII	Sigma	Cat# C2139
Dnase I	Sigma	Cat# 11284932001
Human SCF	Peprotech	Cat# 300–07
Murine SCF	Peprotech	Cat# 250-03
Murine IL-3	Peprotech	Cat# 213-13
SP ELISA Kit	BBI Life Science	Cat# D751030

Table S2 List of DNA primer sequences for qRT-PCR

Target gene	Forward 5’-3’	Reverse 5’-3’
Tac1	TTTCTCGTTTCCACTCAACTGTT	GTCTTCGGGCGATTCTCTGC
Tnfa	AGCAGAAGCTCCCTCAGCGAGG	TCCACGTCGCGGATCATGCTTT
Il1b	GGAGAACCAAGCAACGACAAAATA	TGGGGAACTCTGCAGACTCAAAC
Cxcl1	ATCCAGAGCTTGAAGGTGTTG	GTCTGTCTTCTTTCTCCGTTACTT
Sphk1	CGTGGACCTCGAGAGTGAGAA	AGGCTTGCTAGGCGAAAGAAG
Actb	CTGAATGGCCCAGGTCTGA	CCCTGGCTGCCTCAACAC

49 Spadina Ave. Suite 200
Toronto ON M5V 2J1 Canada
www.biorender.com

Confirmation of Publication and Licensing Rights - Open Access

December 1st, 2025

Subscription Type:	*Individual - Academic*
Agreement number:	*ZC292ANXLU*
Publisher Name:	*JCI Insight*
Figure Title:	*Fig. 1A*
Citation to Use:	*Created in BioRender. Qu, L. (2026) https://BioRender.com/jh5fc7d*

To whom this may concern,

This document ("Confirmation") hereby confirms that Science Suite Inc. dba BioRender ("BioRender") has granted the following BioRender user: Lintao Qu ("User") a BioRender Academic Publication License in accordance with BioRender's Terms of Service and Academic License Terms ("License Terms") to permit such User to do the following on the condition that all requirements in this Confirmation are met:

1) publish their Completed Graphics created in the BioRender Services containing both User Content and BioRender Content (as both are defined in the License Terms) in publications (journals, textbooks, websites, etc.); and
2) sublicense such Completed Graphics under "open access" publication sublicensing models such as CC-BY 4.0 and more restrictive models, so long as the conditions set forth herein are fully met.

Requirements of User:

1) All Completed Graphics to be published in any publication (journals, textbooks, websites, etc.) must be accompanied by the following citation either as a caption, footnote or reference for each figure that includes a Completed Graphic:
"Created in BioRender. Qu, L. (2026) https://BioRender.com/jh5fc7d ".
2) All terms of the License Terms including all Prohibited Uses are fully complied with. E.g. For Academic License Users, no commercial uses (beyond publication in journals, textbooks or websites) are permitted without obtaining or switching to a BioRender Industry Plan.
3) A Reader (defined below) may request that the User allow their figure to be a public template for Readers to view, copy, and modify the figure. It is up to the User to determine what level of access to grant.

Open-Access Journal Readers:

Open-Access journal readers ("Reader") who wish to view and/or re-use a particular Completed Graphic in an Open-Access journal subject to CC-BY sublicensing may do so by clicking on the URL link in the applicable citation for the subject Completed Graphic.

The re-use/modification options below are available after the Reader requests the User to adapt their

figure as a BioRender template and the User has granted such access.

1) View-Only/Free Plan Use: A Reader who wishes to only view the Completed Graphic may do so in the BioRender Services as either a BioRender Free Plan user or simply as a viewer. By becoming a BioRender Free Plan user, the Reader may view, modify and re-use the Completed Graphic as permitted under BioRender's Basic License Terms (e.g. personal use only, no publishing or commercial use permitted).
2) Re-Use/Publish with No Modifications: For any re-use and re-publication of a Completed Graphic with no modification(s) to the Completed Graphic made by the Reader, a Reader may do so by citing the original author using the citation noted above with the Completed Graphic. The Reader must also comply with the underlying License Terms which apply to the Completed Graphic as noted above (e.g. no commercial use for Academic License).
3) Re-Use/Publish with Modifications: For any re-use and re-publication of a Completed Graphic with a modification(s) made by the Reader, the Reader may do so by becoming a BioRender user themselves under either an Academic or Industry Plan, citing the original author using the citation noted above with the Completed Graphic and complying with the applicable License Terms.

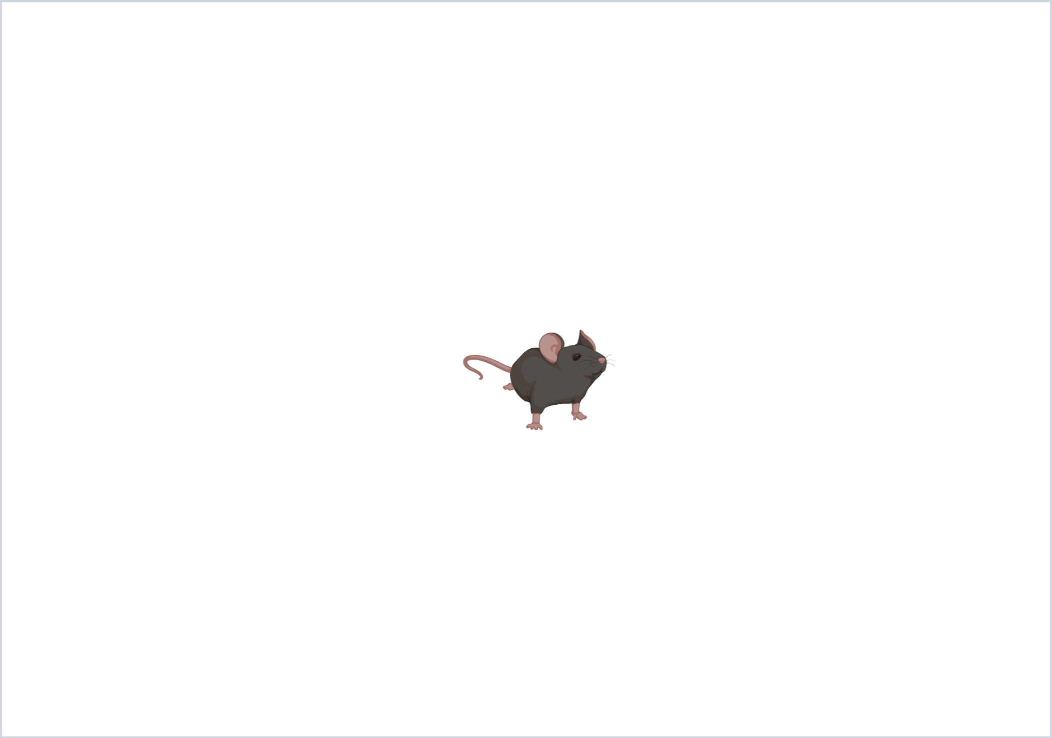

For any questions regarding this document, or other questions about publishing with BioRender, please refer to our BioRender Publication Guide, or contact BioRender Support at support@biorender.com.

49 Spadina Ave. Suite 200
Toronto ON M5V 2J1 Canada
www.biorender.com

Confirmation of Publication and Licensing Rights - Open Access

December 1st, 2025

Subscription Type:	*Individual - Academic*
Agreement number:	*XM292AOVAY*
Publisher Name:	*JCI Insight*
Figure Title:	*Fig. 1A*
Citation to Use:	*Created in BioRender. Qu, L. (2026) https://BioRender.com/jh5fc7d*

To whom this may concern,

This document ("Confirmation") hereby confirms that Science Suite Inc. dba BioRender ("BioRender") has granted the following BioRender user: Lintao Qu ("User") a BioRender Academic Publication License in accordance with BioRender's Terms of Service and Academic License Terms ("License Terms") to permit such User to do the following on the condition that all requirements in this Confirmation are met:

1) publish their Completed Graphics created in the BioRender Services containing both User Content and BioRender Content (as both are defined in the License Terms) in publications (journals, textbooks, websites, etc.); and
2) sublicense such Completed Graphics under "open access" publication sublicensing models such as CC-BY 4.0 and more restrictive models, so long as the conditions set forth herein are fully met.

Requirements of User:

1) All Completed Graphics to be published in any publication (journals, textbooks, websites, etc.) must be accompanied by the following citation either as a caption, footnote or reference for each figure that includes a Completed Graphic:
"Created in BioRender. Qu, L. (2026) https://BioRender.com/jh5fc7d ".
2) All terms of the License Terms including all Prohibited Uses are fully complied with. E.g. For Academic License Users, no commercial uses (beyond publication in journals, textbooks or websites) are permitted without obtaining or switching to a BioRender Industry Plan.
3) A Reader (defined below) may request that the User allow their figure to be a public template for Readers to view, copy, and modify the figure. It is up to the User to determine what level of access to grant.

Open-Access Journal Readers:

Open-Access journal readers ("Reader") who wish to view and/or re-use a particular Completed Graphic in an Open-Access journal subject to CC-BY sublicensing may do so by clicking on the URL link in the applicable citation for the subject Completed Graphic.

The re-use/modification options below are available after the Reader requests the User to adapt their

figure as a BioRender template and the User has granted such access.

1) View-Only/Free Plan Use: A Reader who wishes to only view the Completed Graphic may do so in the BioRender Services as either a BioRender Free Plan user or simply as a viewer. By becoming a BioRender Free Plan user, the Reader may view, modify and re-use the Completed Graphic as permitted under BioRender's Basic License Terms (e.g. personal use only, no publishing or commercial use permitted).
2) Re-Use/Publish with No Modifications: For any re-use and re-publication of a Completed Graphic with no modification(s) to the Completed Graphic made by the Reader, a Reader may do so by citing the original author using the citation noted above with the Completed Graphic. The Reader must also comply with the underlying License Terms which apply to the Completed Graphic as noted above (e.g. no commercial use for Academic License).
3) Re-Use/Publish with Modifications: For any re-use and re-publication of a Completed Graphic with a modification(s) made by the Reader, the Reader may do so by becoming a BioRender user themselves under either an Academic or Industry Plan, citing the original author using the citation noted above with the Completed Graphic and complying with the applicable License Terms.

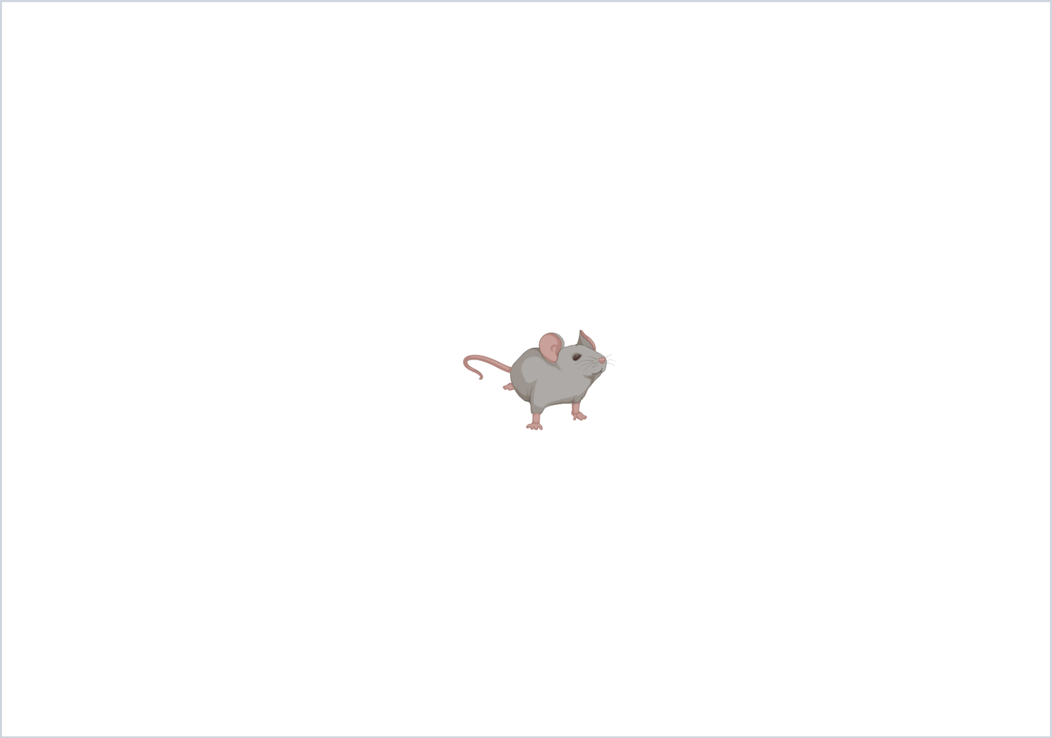

For any questions regarding this document, or other questions about publishing with BioRender, please refer to our BioRender Publication Guide, or contact BioRender Support at support@biorender.com.

49 Spadina Ave. Suite 200
Toronto ON M5V 2J1 Canada
www.biorender.com

Confirmation of Publication and Licensing Rights - Open Access

December 1st, 2025

Subscription Type:	*Individual - Academic*
Agreement number:	*SD292APHK7*
Publisher Name:	*JCI Insight*
Figure Title:	*Fig. 1A*
Citation to Use:	*Created in BioRender. Qu, L. (2026) https://BioRender.com/jh5fc7d*

To whom this may concern,

This document ("Confirmation") hereby confirms that Science Suite Inc. dba BioRender ("BioRender") has granted the following BioRender user: Lintao Qu ("User") a BioRender Academic Publication License in accordance with BioRender's Terms of Service and Academic License Terms ("License Terms") to permit such User to do the following on the condition that all requirements in this Confirmation are met:

1) publish their Completed Graphics created in the BioRender Services containing both User Content and BioRender Content (as both are defined in the License Terms) in publications (journals, textbooks, websites, etc.); and
2) sublicense such Completed Graphics under "open access" publication sublicensing models such as CC-BY 4.0 and more restrictive models, so long as the conditions set forth herein are fully met.

Requirements of User:

1) All Completed Graphics to be published in any publication (journals, textbooks, websites, etc.) must be accompanied by the following citation either as a caption, footnote or reference for each figure that includes a Completed Graphic:
"Created in BioRender. Qu, L. (2026) https://BioRender.com/jh5fc7d ".
2) All terms of the License Terms including all Prohibited Uses are fully complied with. E.g. For Academic License Users, no commercial uses (beyond publication in journals, textbooks or websites) are permitted without obtaining or switching to a BioRender Industry Plan.
3) A Reader (defined below) may request that the User allow their figure to be a public template for Readers to view, copy, and modify the figure. It is up to the User to determine what level of access to grant.

Open-Access Journal Readers:

Open-Access journal readers ("Reader") who wish to view and/or re-use a particular Completed Graphic in an Open-Access journal subject to CC-BY sublicensing may do so by clicking on the URL link in the applicable citation for the subject Completed Graphic.

The re-use/modification options below are available after the Reader requests the User to adapt their

figure as a BioRender template and the User has granted such access.

1) View-Only/Free Plan Use: A Reader who wishes to only view the Completed Graphic may do so in the BioRender Services as either a BioRender Free Plan user or simply as a viewer. By becoming a BioRender Free Plan user, the Reader may view, modify and re-use the Completed Graphic as permitted under BioRender's Basic License Terms (e.g. personal use only, no publishing or commercial use permitted).
2) Re-Use/Publish with No Modifications: For any re-use and re-publication of a Completed Graphic with no modification(s) to the Completed Graphic made by the Reader, a Reader may do so by citing the original author using the citation noted above with the Completed Graphic. The Reader must also comply with the underlying License Terms which apply to the Completed Graphic as noted above (e.g. no commercial use for Academic License).
3) Re-Use/Publish with Modifications: For any re-use and re-publication of a Completed Graphic with a modification(s) made by the Reader, the Reader may do so by becoming a BioRender user themselves under either an Academic or Industry Plan, citing the original author using the citation noted above with the Completed Graphic and complying with the applicable License Terms.

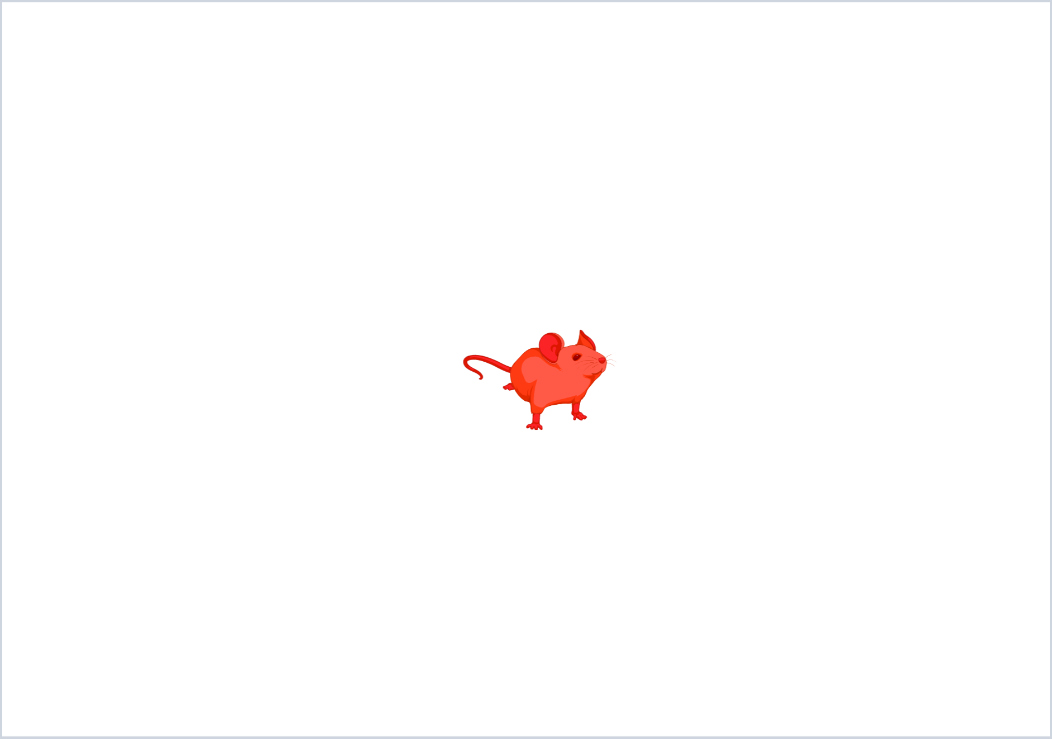

For any questions regarding this document, or other questions about publishing with BioRender, please refer to our BioRender Publication Guide, or contact BioRender Support at support@biorender.com.

49 Spadina Ave. Suite 200
Toronto ON M5V 2J1 Canada
www.biorender.com

Confirmation of Publication and Licensing Rights - Open Access

December 1st, 2025

Subscription Type: *Individual - Academic*
Agreement number: *FC292AQAD6*
Publisher Name: *JCI Insight*

Figure Title: *Fig. 3A*

Citation to Use: *Created in BioRender. Qu, L. (2026) https://BioRender.com/0i7869s*

To whom this may concern,

This document ("Confirmation") hereby confirms that Science Suite Inc. dba BioRender ("BioRender") has granted the following BioRender user: Lintao Qu ("User") a BioRender Academic Publication License in accordance with BioRender's Terms of Service and Academic License Terms ("License Terms") to permit such User to do the following on the condition that all requirements in this Confirmation are met:

1) publish their Completed Graphics created in the BioRender Services containing both User Content and BioRender Content (as both are defined in the License Terms) in publications (journals, textbooks, websites, etc.); and
2) sublicense such Completed Graphics under "open access" publication sublicensing models such as CC-BY 4.0 and more restrictive models, so long as the conditions set forth herein are fully met.

Requirements of User:

1) All Completed Graphics to be published in any publication (journals, textbooks, websites, etc.) must be accompanied by the following citation either as a caption, footnote or reference for each figure that includes a Completed Graphic:
"Created in BioRender. Qu, L. (2026) https://BioRender.com/0i7869s ".
2) All terms of the License Terms including all Prohibited Uses are fully complied with. E.g. For Academic License Users, no commercial uses (beyond publication in journals, textbooks or websites) are permitted without obtaining or switching to a BioRender Industry Plan.
3) A Reader (defined below) may request that the User allow their figure to be a public template for Readers to view, copy, and modify the figure. It is up to the User to determine what level of access to grant.

Open-Access Journal Readers:

Open-Access journal readers ("Reader") who wish to view and/or re-use a particular Completed Graphic in an Open-Access journal subject to CC-BY sublicensing may do so by clicking on the URL link in the applicable citation for the subject Completed Graphic.

The re-use/modification options below are available after the Reader requests the User to adapt their

figure as a BioRender template and the User has granted such access.

1) View-Only/Free Plan Use: A Reader who wishes to only view the Completed Graphic may do so in the BioRender Services as either a BioRender Free Plan user or simply as a viewer. By becoming a BioRender Free Plan user, the Reader may view, modify and re-use the Completed Graphic as permitted under BioRender's Basic License Terms (e.g. personal use only, no publishing or commercial use permitted).
2) Re-Use/Publish with No Modifications: For any re-use and re-publication of a Completed Graphic with no modification(s) to the Completed Graphic made by the Reader, a Reader may do so by citing the original author using the citation noted above with the Completed Graphic. The Reader must also comply with the underlying License Terms which apply to the Completed Graphic as noted above (e.g. no commercial use for Academic License).
3) Re-Use/Publish with Modifications: For any re-use and re-publication of a Completed Graphic with a modification(s) made by the Reader, the Reader may do so by becoming a BioRender user themselves under either an Academic or Industry Plan, citing the original author using the citation noted above with the Completed Graphic and complying with the applicable License Terms.

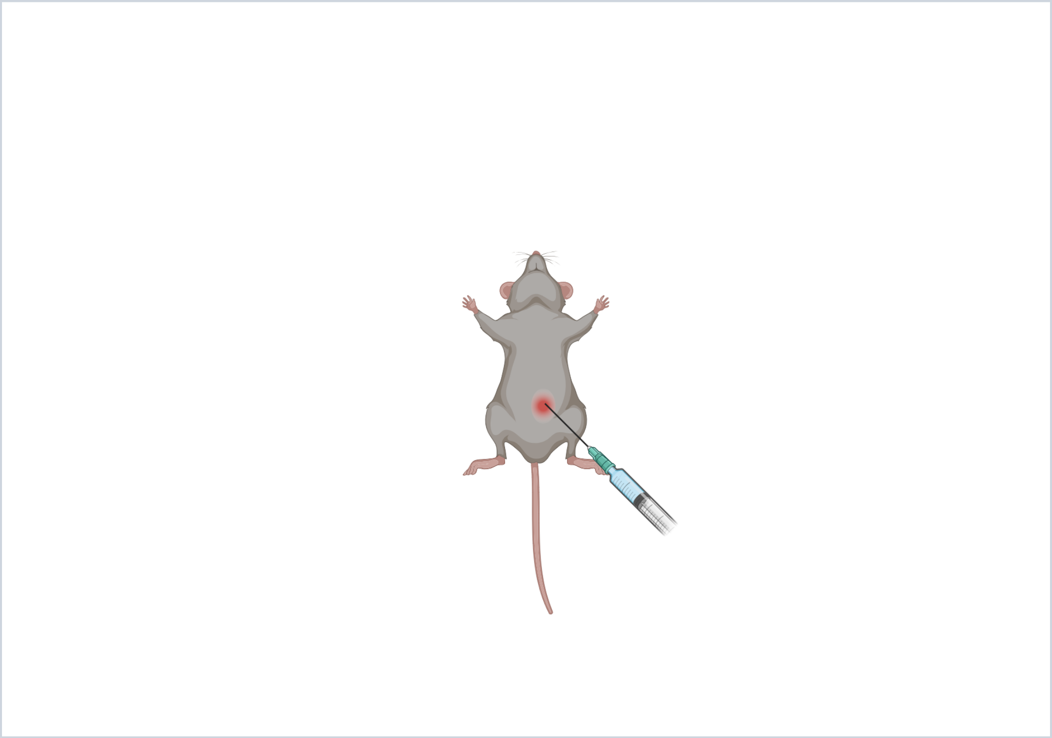

For any questions regarding this document, or other questions about publishing with BioRender, please refer to our BioRender Publication Guide, or contact BioRender Support at support@biorender.com.

49 Spadina Ave. Suite 200
Toronto ON M5V 2J1 Canada
www.biorender.com

Confirmation of Publication and Licensing Rights - Open Access

December 1st, 2025

Subscription Type:	*Individual - Academic*
Agreement number:	*FG292AQW12*
Publisher Name:	*JCI Insight*
Figure Title:	*Fig. 5A*
Citation to Use:	*Created in BioRender. Qu, L. (2026) https://BioRender.com/snnmb6q*

To whom this may concern,

This document ("Confirmation") hereby confirms that Science Suite Inc. dba BioRender ("BioRender") has granted the following BioRender user: Lintao Qu ("User") a BioRender Academic Publication License in accordance with BioRender's Terms of Service and Academic License Terms ("License Terms") to permit such User to do the following on the condition that all requirements in this Confirmation are met:

1) publish their Completed Graphics created in the BioRender Services containing both User Content and BioRender Content (as both are defined in the License Terms) in publications (journals, textbooks, websites, etc.); and
2) sublicense such Completed Graphics under "open access" publication sublicensing models such as CC-BY 4.0 and more restrictive models, so long as the conditions set forth herein are fully met.

Requirements of User:

1) All Completed Graphics to be published in any publication (journals, textbooks, websites, etc.) must be accompanied by the following citation either as a caption, footnote or reference for each figure that includes a Completed Graphic:
"Created in BioRender. Qu, L. (2026) https://BioRender.com/snnmb6q ".
2) All terms of the License Terms including all Prohibited Uses are fully complied with. E.g. For Academic License Users, no commercial uses (beyond publication in journals, textbooks or websites) are permitted without obtaining or switching to a BioRender Industry Plan.
3) A Reader (defined below) may request that the User allow their figure to be a public template for Readers to view, copy, and modify the figure. It is up to the User to determine what level of access to grant.

Open-Access Journal Readers:

Open-Access journal readers ("Reader") who wish to view and/or re-use a particular Completed Graphic in an Open-Access journal subject to CC-BY sublicensing may do so by clicking on the URL link in the applicable citation for the subject Completed Graphic.

The re-use/modification options below are available after the Reader requests the User to adapt their

figure as a BioRender template and the User has granted such access.

1) View-Only/Free Plan Use: A Reader who wishes to only view the Completed Graphic may do so in the BioRender Services as either a BioRender Free Plan user or simply as a viewer. By becoming a BioRender Free Plan user, the Reader may view, modify and re-use the Completed Graphic as permitted under BioRender's Basic License Terms (e.g. personal use only, no publishing or commercial use permitted).
2) Re-Use/Publish with No Modifications: For any re-use and re-publication of a Completed Graphic with no modification(s) to the Completed Graphic made by the Reader, a Reader may do so by citing the original author using the citation noted above with the Completed Graphic. The Reader must also comply with the underlying License Terms which apply to the Completed Graphic as noted above (e.g. no commercial use for Academic License).
3) Re-Use/Publish with Modifications: For any re-use and re-publication of a Completed Graphic with a modification(s) made by the Reader, the Reader may do so by becoming a BioRender user themselves under either an Academic or Industry Plan, citing the original author using the citation noted above with the Completed Graphic and complying with the applicable License Terms.

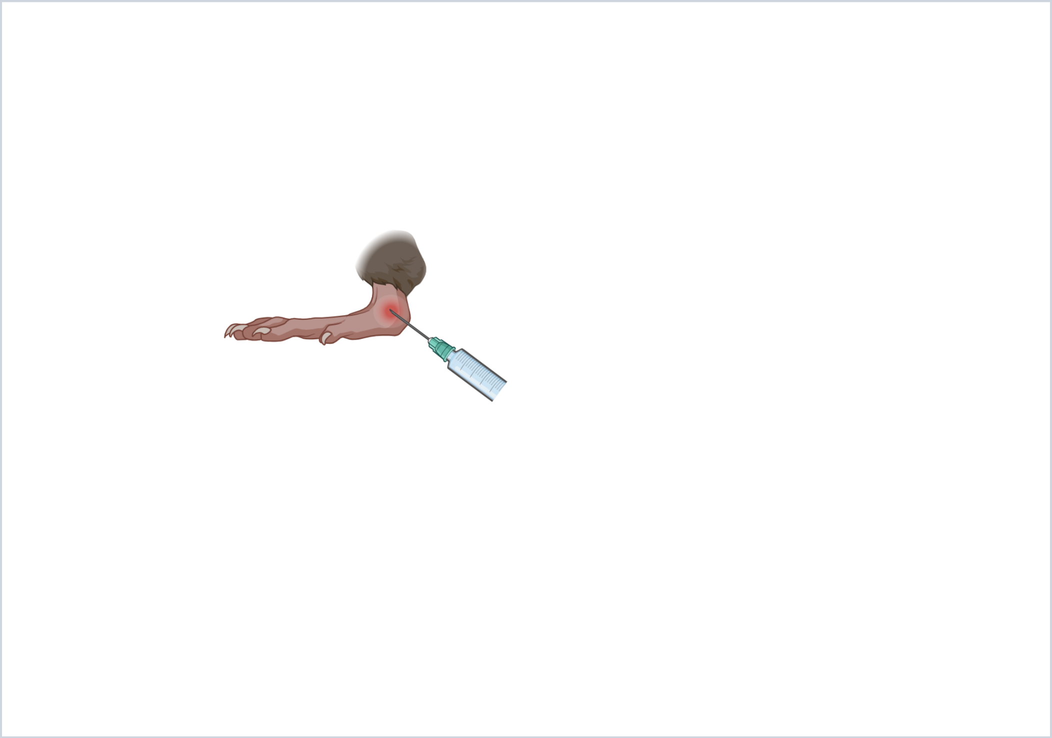

For any questions regarding this document, or other questions about publishing with BioRender, please refer to our BioRender Publication Guide, or contact BioRender Support at support@biorender.com.

49 Spadina Ave. Suite 200
Toronto ON M5V 2J1 Canada
www.biorender.com

Confirmation of Publication and Licensing Rights - Open Access

December 1st, 2025

Subscription Type: *Individual - Academic*
Agreement number: *LD292ARHJ2*
Publisher Name: *JCI Insight*

Figure Title: *Fig. 5F*

Citation to Use: *Created in BioRender. Qu, L. (2026) https://BioRender.com/t0h8dej*

To whom this may concern,

This document ("Confirmation") hereby confirms that Science Suite Inc. dba BioRender ("BioRender") has granted the following BioRender user: Lintao Qu ("User") a BioRender Academic Publication License in accordance with BioRender's Terms of Service and Academic License Terms ("License Terms") to permit such User to do the following on the condition that all requirements in this Confirmation are met:

1) publish their Completed Graphics created in the BioRender Services containing both User Content and BioRender Content (as both are defined in the License Terms) in publications (journals, textbooks, websites, etc.); and
2) sublicense such Completed Graphics under "open access" publication sublicensing models such as CC-BY 4.0 and more restrictive models, so long as the conditions set forth herein are fully met.

Requirements of User:

1) All Completed Graphics to be published in any publication (journals, textbooks, websites, etc.) must be accompanied by the following citation either as a caption, footnote or reference for each figure that includes a Completed Graphic:
"Created in BioRender. Qu, L. (2026) https://BioRender.com/t0h8dej ".
2) All terms of the License Terms including all Prohibited Uses are fully complied with. E.g. For Academic License Users, no commercial uses (beyond publication in journals, textbooks or websites) are permitted without obtaining or switching to a BioRender Industry Plan.
3) A Reader (defined below) may request that the User allow their figure to be a public template for Readers to view, copy, and modify the figure. It is up to the User to determine what level of access to grant.

Open-Access Journal Readers:

Open-Access journal readers ("Reader") who wish to view and/or re-use a particular Completed Graphic in an Open-Access journal subject to CC-BY sublicensing may do so by clicking on the URL link in the applicable citation for the subject Completed Graphic.

The re-use/modification options below are available after the Reader requests the User to adapt their

figure as a BioRender template and the User has granted such access.

1) View-Only/Free Plan Use: A Reader who wishes to only view the Completed Graphic may do so in the BioRender Services as either a BioRender Free Plan user or simply as a viewer. By becoming a BioRender Free Plan user, the Reader may view, modify and re-use the Completed Graphic as permitted under BioRender's Basic License Terms (e.g. personal use only, no publishing or commercial use permitted).
2) Re-Use/Publish with No Modifications: For any re-use and re-publication of a Completed Graphic with no modification(s) to the Completed Graphic made by the Reader, a Reader may do so by citing the original author using the citation noted above with the Completed Graphic. The Reader must also comply with the underlying License Terms which apply to the Completed Graphic as noted above (e.g. no commercial use for Academic License).
3) Re-Use/Publish with Modifications: For any re-use and re-publication of a Completed Graphic with a modification(s) made by the Reader, the Reader may do so by becoming a BioRender user themselves under either an Academic or Industry Plan, citing the original author using the citation noted above with the Completed Graphic and complying with the applicable License Terms.

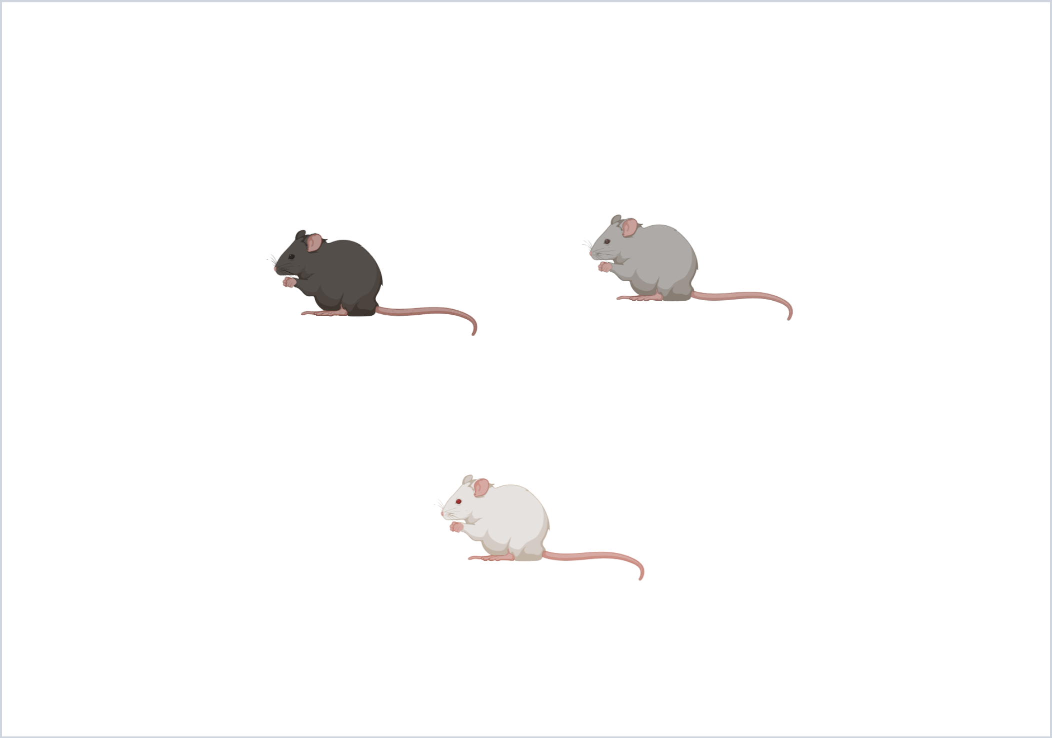

For any questions regarding this document, or other questions about publishing with BioRender, please refer to our BioRender Publication Guide, or contact BioRender Support at support@biorender.com.

49 Spadina Ave. Suite 200
Toronto ON M5V 2J1 Canada
www.biorender.com

Confirmation of Publication and Licensing Rights - Open Access

December 1st, 2025

Subscription Type: *Individual - Academic*
Agreement number: *SQ292AS4F5*
Publisher Name: *JCI Insight*

Figure Title: *Fig. 6A*

Citation to Use: *Created in BioRender. Qu, L. (2026) https://BioRender.com/cdliw1r*

To whom this may concern,

This document ("Confirmation") hereby confirms that Science Suite Inc. dba BioRender ("BioRender") has granted the following BioRender user: Lintao Qu ("User") a BioRender Academic Publication License in accordance with BioRender's Terms of Service and Academic License Terms ("License Terms") to permit such User to do the following on the condition that all requirements in this Confirmation are met:

1) publish their Completed Graphics created in the BioRender Services containing both User Content and BioRender Content (as both are defined in the License Terms) in publications (journals, textbooks, websites, etc.); and
2) sublicense such Completed Graphics under "open access" publication sublicensing models such as CC-BY 4.0 and more restrictive models, so long as the conditions set forth herein are fully met.

Requirements of User:

1) All Completed Graphics to be published in any publication (journals, textbooks, websites, etc.) must be accompanied by the following citation either as a caption, footnote or reference for each figure that includes a Completed Graphic:
"Created in BioRender. Qu, L. (2026) https://BioRender.com/cdliw1r ".
2) All terms of the License Terms including all Prohibited Uses are fully complied with. E.g. For Academic License Users, no commercial uses (beyond publication in journals, textbooks or websites) are permitted without obtaining or switching to a BioRender Industry Plan.
3) A Reader (defined below) may request that the User allow their figure to be a public template for Readers to view, copy, and modify the figure. It is up to the User to determine what level of access to grant.

Open-Access Journal Readers:

Open-Access journal readers ("Reader") who wish to view and/or re-use a particular Completed Graphic in an Open-Access journal subject to CC-BY sublicensing may do so by clicking on the URL link in the applicable citation for the subject Completed Graphic.

The re-use/modification options below are available after the Reader requests the User to adapt their

figure as a BioRender template and the User has granted such access.

1) View-Only/Free Plan Use: A Reader who wishes to only view the Completed Graphic may do so in the BioRender Services as either a BioRender Free Plan user or simply as a viewer. By becoming a BioRender Free Plan user, the Reader may view, modify and re-use the Completed Graphic as permitted under BioRender's Basic License Terms (e.g. personal use only, no publishing or commercial use permitted).
2) Re-Use/Publish with No Modifications: For any re-use and re-publication of a Completed Graphic with no modification(s) to the Completed Graphic made by the Reader, a Reader may do so by citing the original author using the citation noted above with the Completed Graphic. The Reader must also comply with the underlying License Terms which apply to the Completed Graphic as noted above (e.g. no commercial use for Academic License).
3) Re-Use/Publish with Modifications: For any re-use and re-publication of a Completed Graphic with a modification(s) made by the Reader, the Reader may do so by becoming a BioRender user themselves under either an Academic or Industry Plan, citing the original author using the citation noted above with the Completed Graphic and complying with the applicable License Terms.

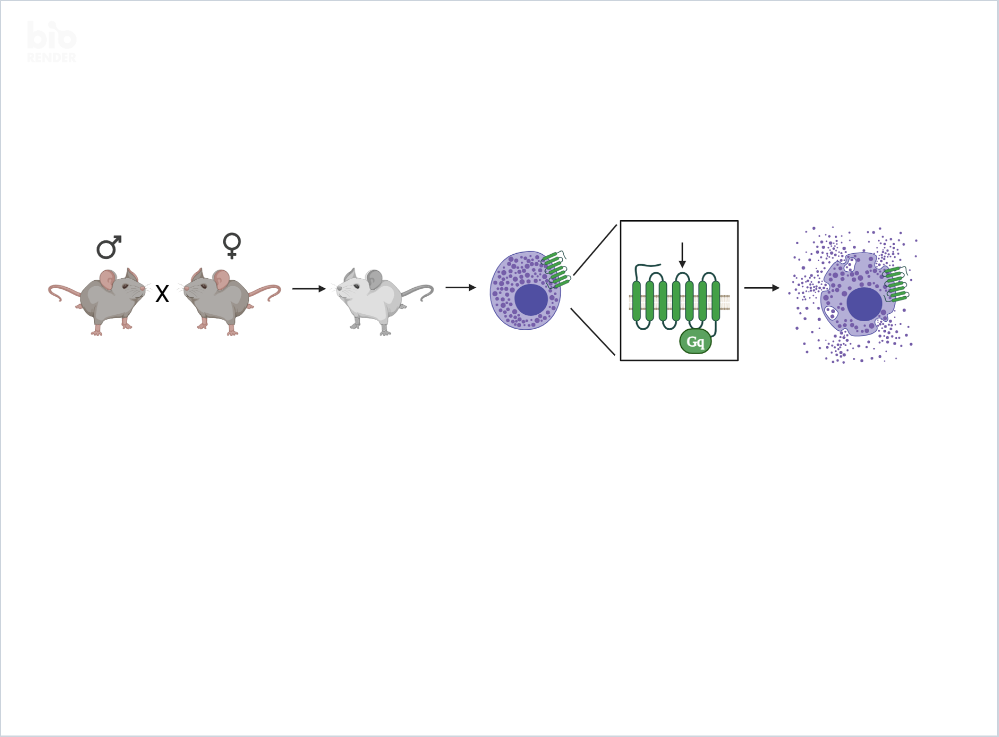

For any questions regarding this document, or other questions about publishing with BioRender, please refer to our BioRender Publication Guide, or contact BioRender Support at support@biorender.com.

49 Spadina Ave. Suite 200
Toronto ON M5V 2J1 Canada
www.biorender.com

Confirmation of Publication and Licensing Rights - Open Access

December 3rd, 2025

Subscription Type: *Individual - Academic*
Agreement number: *NE292J79BV*
Publisher Name: *JCI Insight*

Figure Title: *Fig. 6E*

Citation to Use: *Created in BioRender. Qu, L. (2026) https://BioRender.com/dyysb9x*

To whom this may concern,

This document ("Confirmation") hereby confirms that Science Suite Inc. dba BioRender ("BioRender") has granted the following BioRender user: Lintao Qu ("User") a BioRender Academic Publication License in accordance with BioRender's Terms of Service and Academic License Terms ("License Terms") to permit such User to do the following on the condition that all requirements in this Confirmation are met:

1) publish their Completed Graphics created in the BioRender Services containing both User Content and BioRender Content (as both are defined in the License Terms) in publications (journals, textbooks, websites, etc.); and
2) sublicense such Completed Graphics under "open access" publication sublicensing models such as CC-BY 4.0 and more restrictive models, so long as the conditions set forth herein are fully met.

Requirements of User:

1) All Completed Graphics to be published in any publication (journals, textbooks, websites, etc.) must be accompanied by the following citation either as a caption, footnote or reference for each figure that includes a Completed Graphic:
"Created in BioRender. Qu, L. (2026) https://BioRender.com/dyysb9x ".
2) All terms of the License Terms including all Prohibited Uses are fully complied with. E.g. For Academic License Users, no commercial uses (beyond publication in journals, textbooks or websites) are permitted without obtaining or switching to a BioRender Industry Plan.
3) A Reader (defined below) may request that the User allow their figure to be a public template for Readers to view, copy, and modify the figure. It is up to the User to determine what level of access to grant.

Open-Access Journal Readers:

Open-Access journal readers ("Reader") who wish to view and/or re-use a particular Completed Graphic in an Open-Access journal subject to CC-BY sublicensing may do so by clicking on the URL link in the applicable citation for the subject Completed Graphic.

The re-use/modification options below are available after the Reader requests the User to adapt their

figure as a BioRender template and the User has granted such access.

1) View-Only/Free Plan Use: A Reader who wishes to only view the Completed Graphic may do so in the BioRender Services as either a BioRender Free Plan user or simply as a viewer. By becoming a BioRender Free Plan user, the Reader may view, modify and re-use the Completed Graphic as permitted under BioRender's Basic License Terms (e.g. personal use only, no publishing or commercial use permitted).
2) Re-Use/Publish with No Modifications: For any re-use and re-publication of a Completed Graphic with no modification(s) to the Completed Graphic made by the Reader, a Reader may do so by citing the original author using the citation noted above with the Completed Graphic. The Reader must also comply with the underlying License Terms which apply to the Completed Graphic as noted above (e.g. no commercial use for Academic License).
3) Re-Use/Publish with Modifications: For any re-use and re-publication of a Completed Graphic with a modification(s) made by the Reader, the Reader may do so by becoming a BioRender user themselves under either an Academic or Industry Plan, citing the original author using the citation noted above with the Completed Graphic and complying with the applicable License Terms.

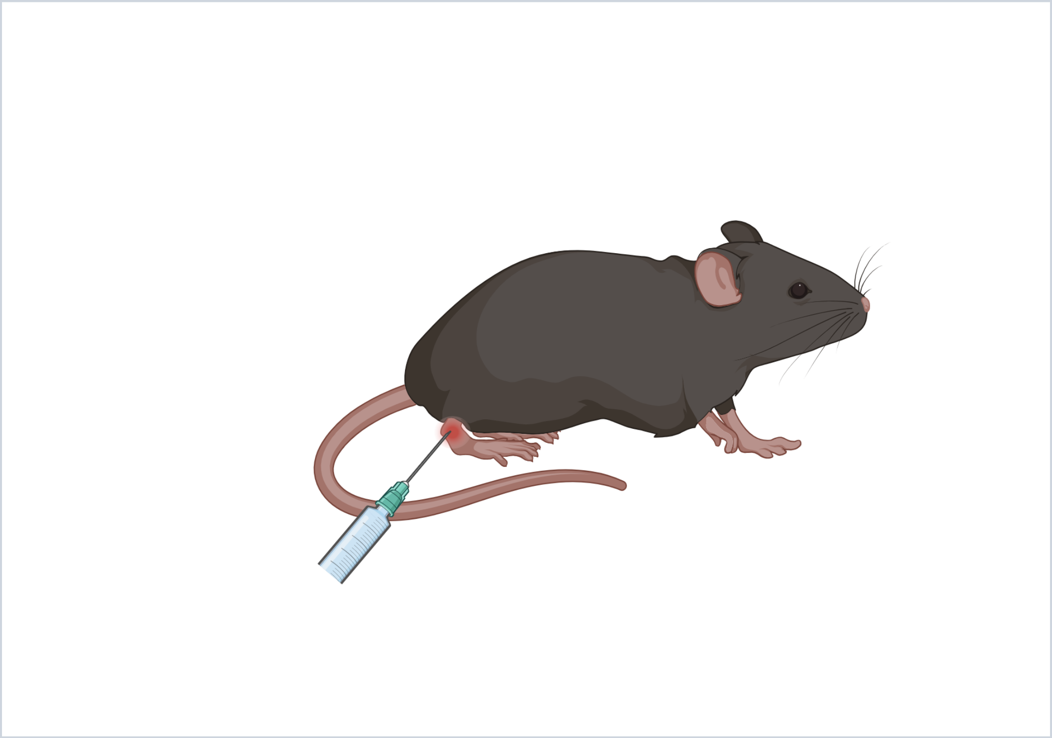

For any questions regarding this document, or other questions about publishing with BioRender, please refer to our BioRender Publication Guide, or contact BioRender Support at support@biorender.com.

49 Spadina Ave. Suite 200
Toronto ON M5V 2J1 Canada
www.biorender.com

Confirmation of Publication and Licensing Rights - Open Access

December 1st, 2025

Subscription Type:	*Individual - Academic*
Agreement number:	*XJ292ASX61*
Publisher Name:	*JCI Insight*
Figure Title:	*Fig. 7A*
Citation to Use:	*Created in BioRender. Qu, L. (2026) https://BioRender.com/tvek5ah*

To whom this may concern,

This document ("Confirmation") hereby confirms that Science Suite Inc. dba BioRender ("BioRender") has granted the following BioRender user: Lintao Qu ("User") a BioRender Academic Publication License in accordance with BioRender's Terms of Service and Academic License Terms ("License Terms") to permit such User to do the following on the condition that all requirements in this Confirmation are met:

1) publish their Completed Graphics created in the BioRender Services containing both User Content and BioRender Content (as both are defined in the License Terms) in publications (journals, textbooks, websites, etc.); and
2) sublicense such Completed Graphics under "open access" publication sublicensing models such as CC-BY 4.0 and more restrictive models, so long as the conditions set forth herein are fully met.

Requirements of User:

1) All Completed Graphics to be published in any publication (journals, textbooks, websites, etc.) must be accompanied by the following citation either as a caption, footnote or reference for each figure that includes a Completed Graphic:
"Created in BioRender. Qu, L. (2026) https://BioRender.com/tvek5ah ".
2) All terms of the License Terms including all Prohibited Uses are fully complied with. E.g. For Academic License Users, no commercial uses (beyond publication in journals, textbooks or websites) are permitted without obtaining or switching to a BioRender Industry Plan.
3) A Reader (defined below) may request that the User allow their figure to be a public template for Readers to view, copy, and modify the figure. It is up to the User to determine what level of access to grant.

Open-Access Journal Readers:

Open-Access journal readers ("Reader") who wish to view and/or re-use a particular Completed Graphic in an Open-Access journal subject to CC-BY sublicensing may do so by clicking on the URL link in the applicable citation for the subject Completed Graphic.

The re-use/modification options below are available after the Reader requests the User to adapt their

figure as a BioRender template and the User has granted such access.

1) View-Only/Free Plan Use: A Reader who wishes to only view the Completed Graphic may do so in the BioRender Services as either a BioRender Free Plan user or simply as a viewer. By becoming a BioRender Free Plan user, the Reader may view, modify and re-use the Completed Graphic as permitted under BioRender's Basic License Terms (e.g. personal use only, no publishing or commercial use permitted).
2) Re-Use/Publish with No Modifications: For any re-use and re-publication of a Completed Graphic with no modification(s) to the Completed Graphic made by the Reader, a Reader may do so by citing the original author using the citation noted above with the Completed Graphic. The Reader must also comply with the underlying License Terms which apply to the Completed Graphic as noted above (e.g. no commercial use for Academic License).
3) Re-Use/Publish with Modifications: For any re-use and re-publication of a Completed Graphic with a modification(s) made by the Reader, the Reader may do so by becoming a BioRender user themselves under either an Academic or Industry Plan, citing the original author using the citation noted above with the Completed Graphic and complying with the applicable License Terms.

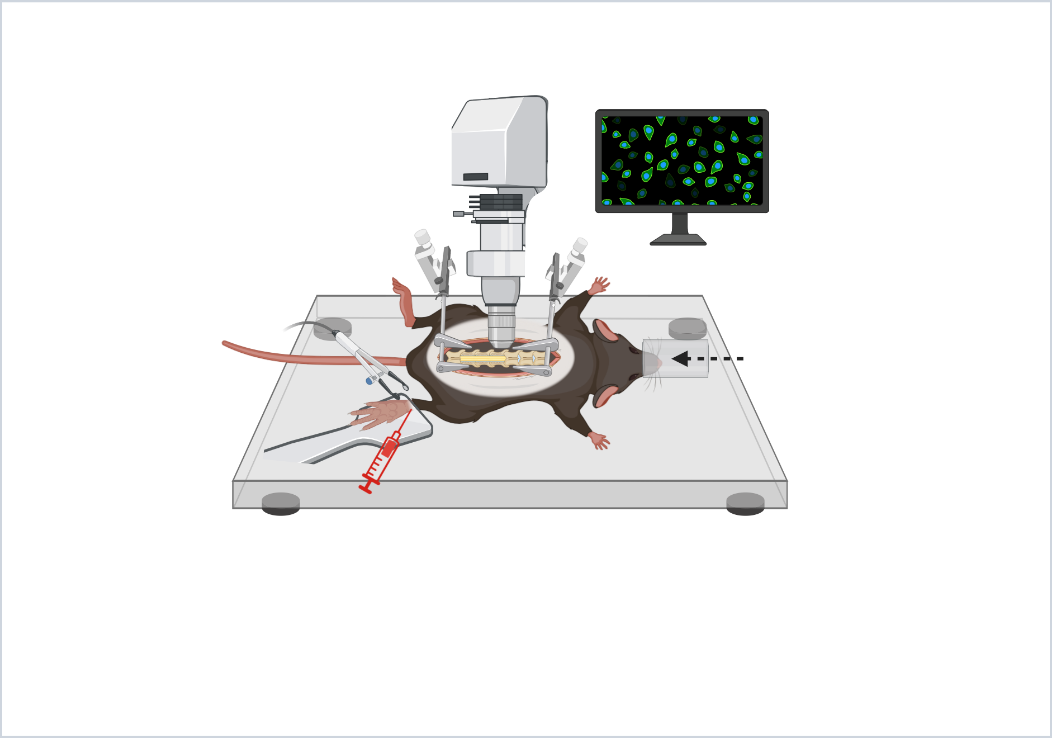

For any questions regarding this document, or other questions about publishing with BioRender, please refer to our BioRender Publication Guide, or contact BioRender Support at support@biorender.com.

49 Spadina Ave. Suite 200
Toronto ON M5V 2J1 Canada
www.biorender.com

Confirmation of Publication and Licensing Rights - Open Access

December 1st, 2025

Subscription Type: *Individual - Academic*
Agreement number: *CW292ATA7I*
Publisher Name: *JCI Insight*

Figure Title: *Fig. 8D*

Citation to Use: *Created in BioRender. Qu, L. (2026) https://BioRender.com/fyi3u5l*

To whom this may concern,

This document ("Confirmation") hereby confirms that Science Suite Inc. dba BioRender ("BioRender") has granted the following BioRender user: Lintao Qu ("User") a BioRender Academic Publication License in accordance with BioRender's Terms of Service and Academic License Terms ("License Terms") to permit such User to do the following on the condition that all requirements in this Confirmation are met:

1) publish their Completed Graphics created in the BioRender Services containing both User Content and BioRender Content (as both are defined in the License Terms) in publications (journals, textbooks, websites, etc.); and
2) sublicense such Completed Graphics under "open access" publication sublicensing models such as CC-BY 4.0 and more restrictive models, so long as the conditions set forth herein are fully met.

Requirements of User:

1) All Completed Graphics to be published in any publication (journals, textbooks, websites, etc.) must be accompanied by the following citation either as a caption, footnote or reference for each figure that includes a Completed Graphic:
"Created in BioRender. Qu, L. (2026) https://BioRender.com/fyi3u5l ".
2) All terms of the License Terms including all Prohibited Uses are fully complied with. E.g. For Academic License Users, no commercial uses (beyond publication in journals, textbooks or websites) are permitted without obtaining or switching to a BioRender Industry Plan.
3) A Reader (defined below) may request that the User allow their figure to be a public template for Readers to view, copy, and modify the figure. It is up to the User to determine what level of access to grant.

Open-Access Journal Readers:

Open-Access journal readers ("Reader") who wish to view and/or re-use a particular Completed Graphic in an Open-Access journal subject to CC-BY sublicensing may do so by clicking on the URL link in the applicable citation for the subject Completed Graphic.

The re-use/modification options below are available after the Reader requests the User to adapt their

figure as a BioRender template and the User has granted such access.

1) View-Only/Free Plan Use: A Reader who wishes to only view the Completed Graphic may do so in the BioRender Services as either a BioRender Free Plan user or simply as a viewer. By becoming a BioRender Free Plan user, the Reader may view, modify and re-use the Completed Graphic as permitted under BioRender's Basic License Terms (e.g. personal use only, no publishing or commercial use permitted).
2) Re-Use/Publish with No Modifications: For any re-use and re-publication of a Completed Graphic with no modification(s) to the Completed Graphic made by the Reader, a Reader may do so by citing the original author using the citation noted above with the Completed Graphic. The Reader must also comply with the underlying License Terms which apply to the Completed Graphic as noted above (e.g. no commercial use for Academic License).
3) Re-Use/Publish with Modifications: For any re-use and re-publication of a Completed Graphic with a modification(s) made by the Reader, the Reader may do so by becoming a BioRender user themselves under either an Academic or Industry Plan, citing the original author using the citation noted above with the Completed Graphic and complying with the applicable License Terms.

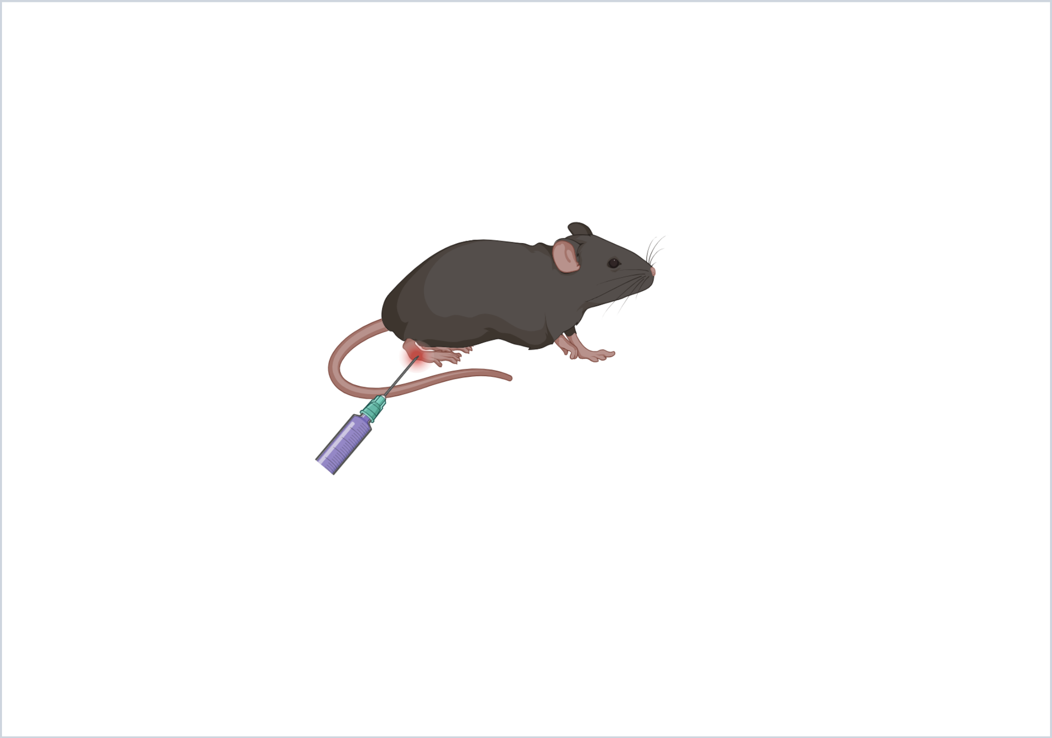

For any questions regarding this document, or other questions about publishing with BioRender, please refer to our BioRender Publication Guide, or contact BioRender Support at support@biorender.com.

49 Spadina Ave. Suite 200
Toronto ON M5V 2J1 Canada
www.biorender.com

Confirmation of Publication and Licensing Rights - Open Access

December 1st, 2025

Subscription Type: *Individual - Academic*
Agreement number: *SK292AUEPV*
Publisher Name: *JCI Insight*

Figure Title: *Fig. 9D*

Citation to Use: *Created in BioRender. Qu, L. (2026) https://BioRender.com/snnmb6q*

To whom this may concern,

This document ("Confirmation") hereby confirms that Science Suite Inc. dba BioRender ("BioRender") has granted the following BioRender user: Lintao Qu ("User") a BioRender Academic Publication License in accordance with BioRender's Terms of Service and Academic License Terms ("License Terms") to permit such User to do the following on the condition that all requirements in this Confirmation are met:

1) publish their Completed Graphics created in the BioRender Services containing both User Content and BioRender Content (as both are defined in the License Terms) in publications (journals, textbooks, websites, etc.); and
2) sublicense such Completed Graphics under "open access" publication sublicensing models such as CC-BY 4.0 and more restrictive models, so long as the conditions set forth herein are fully met.

Requirements of User:

1) All Completed Graphics to be published in any publication (journals, textbooks, websites, etc.) must be accompanied by the following citation either as a caption, footnote or reference for each figure that includes a Completed Graphic:
"Created in BioRender. Qu, L. (2026) https://BioRender.com/snnmb6q ".
2) All terms of the License Terms including all Prohibited Uses are fully complied with. E.g. For Academic License Users, no commercial uses (beyond publication in journals, textbooks or websites) are permitted without obtaining or switching to a BioRender Industry Plan.
3) A Reader (defined below) may request that the User allow their figure to be a public template for Readers to view, copy, and modify the figure. It is up to the User to determine what level of access to grant.

Open-Access Journal Readers:

Open-Access journal readers ("Reader") who wish to view and/or re-use a particular Completed Graphic in an Open-Access journal subject to CC-BY sublicensing may do so by clicking on the URL link in the applicable citation for the subject Completed Graphic.

The re-use/modification options below are available after the Reader requests the User to adapt their

figure as a BioRender template and the User has granted such access.

1) View-Only/Free Plan Use: A Reader who wishes to only view the Completed Graphic may do so in the BioRender Services as either a BioRender Free Plan user or simply as a viewer. By becoming a BioRender Free Plan user, the Reader may view, modify and re-use the Completed Graphic as permitted under BioRender's Basic License Terms (e.g. personal use only, no publishing or commercial use permitted).
2) Re-Use/Publish with No Modifications: For any re-use and re-publication of a Completed Graphic with no modification(s) to the Completed Graphic made by the Reader, a Reader may do so by citing the original author using the citation noted above with the Completed Graphic. The Reader must also comply with the underlying License Terms which apply to the Completed Graphic as noted above (e.g. no commercial use for Academic License).
3) Re-Use/Publish with Modifications: For any re-use and re-publication of a Completed Graphic with a modification(s) made by the Reader, the Reader may do so by becoming a BioRender user themselves under either an Academic or Industry Plan, citing the original author using the citation noted above with the Completed Graphic and complying with the applicable License Terms.

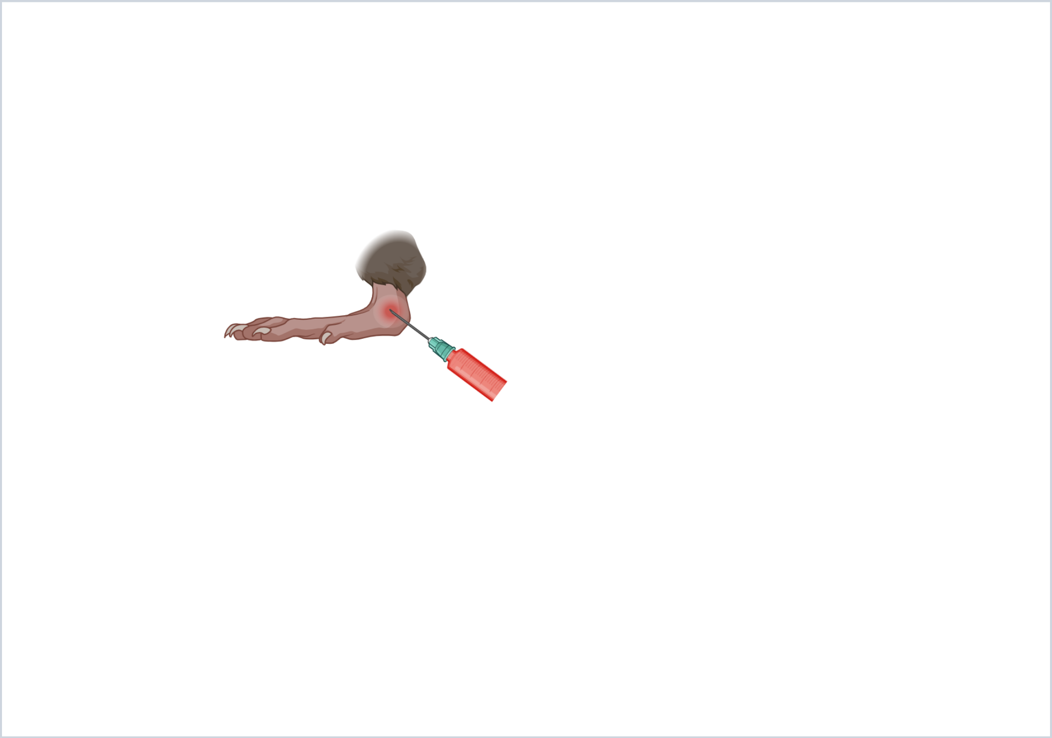

For any questions regarding this document, or other questions about publishing with BioRender, please refer to our BioRender Publication Guide, or contact BioRender Support at support@biorender.com.

49 Spadina Ave. Suite 200
Toronto ON M5V 2J1 Canada
www.biorender.com

Confirmation of Publication and Licensing Rights - Open Access

December 1st, 2025

Subscription Type: *Individual - Academic*
Agreement number: *JZ292AV3A9*
Publisher Name: *JCI Insight*

Figure Title: *Fig. 10A*

Citation to Use: *Created in BioRender. Qu, L. (2026) https://BioRender.com/2xwxwpq*

To whom this may concern,

This document ("Confirmation") hereby confirms that Science Suite Inc. dba BioRender ("BioRender") has granted the following BioRender user: Lintao Qu ("User") a BioRender Academic Publication License in accordance with BioRender's Terms of Service and Academic License Terms ("License Terms") to permit such User to do the following on the condition that all requirements in this Confirmation are met:

1) publish their Completed Graphics created in the BioRender Services containing both User Content and BioRender Content (as both are defined in the License Terms) in publications (journals, textbooks, websites, etc.); and
2) sublicense such Completed Graphics under "open access" publication sublicensing models such as CC-BY 4.0 and more restrictive models, so long as the conditions set forth herein are fully met.

Requirements of User:

1) All Completed Graphics to be published in any publication (journals, textbooks, websites, etc.) must be accompanied by the following citation either as a caption, footnote or reference for each figure that includes a Completed Graphic:
"Created in BioRender. Qu, L. (2026) https://BioRender.com/2xwxwpq ".
2) All terms of the License Terms including all Prohibited Uses are fully complied with. E.g. For Academic License Users, no commercial uses (beyond publication in journals, textbooks or websites) are permitted without obtaining or switching to a BioRender Industry Plan.
3) A Reader (defined below) may request that the User allow their figure to be a public template for Readers to view, copy, and modify the figure. It is up to the User to determine what level of access to grant.

Open-Access Journal Readers:

Open-Access journal readers ("Reader") who wish to view and/or re-use a particular Completed Graphic in an Open-Access journal subject to CC-BY sublicensing may do so by clicking on the URL link in the applicable citation for the subject Completed Graphic.

The re-use/modification options below are available after the Reader requests the User to adapt their

figure as a BioRender template and the User has granted such access.

1) View-Only/Free Plan Use: A Reader who wishes to only view the Completed Graphic may do so in the BioRender Services as either a BioRender Free Plan user or simply as a viewer. By becoming a BioRender Free Plan user, the Reader may view, modify and re-use the Completed Graphic as permitted under BioRender's Basic License Terms (e.g. personal use only, no publishing or commercial use permitted).
2) Re-Use/Publish with No Modifications: For any re-use and re-publication of a Completed Graphic with no modification(s) to the Completed Graphic made by the Reader, a Reader may do so by citing the original author using the citation noted above with the Completed Graphic. The Reader must also comply with the underlying License Terms which apply to the Completed Graphic as noted above (e.g. no commercial use for Academic License).
3) Re-Use/Publish with Modifications: For any re-use and re-publication of a Completed Graphic with a modification(s) made by the Reader, the Reader may do so by becoming a BioRender user themselves under either an Academic or Industry Plan, citing the original author using the citation noted above with the Completed Graphic and complying with the applicable License Terms.

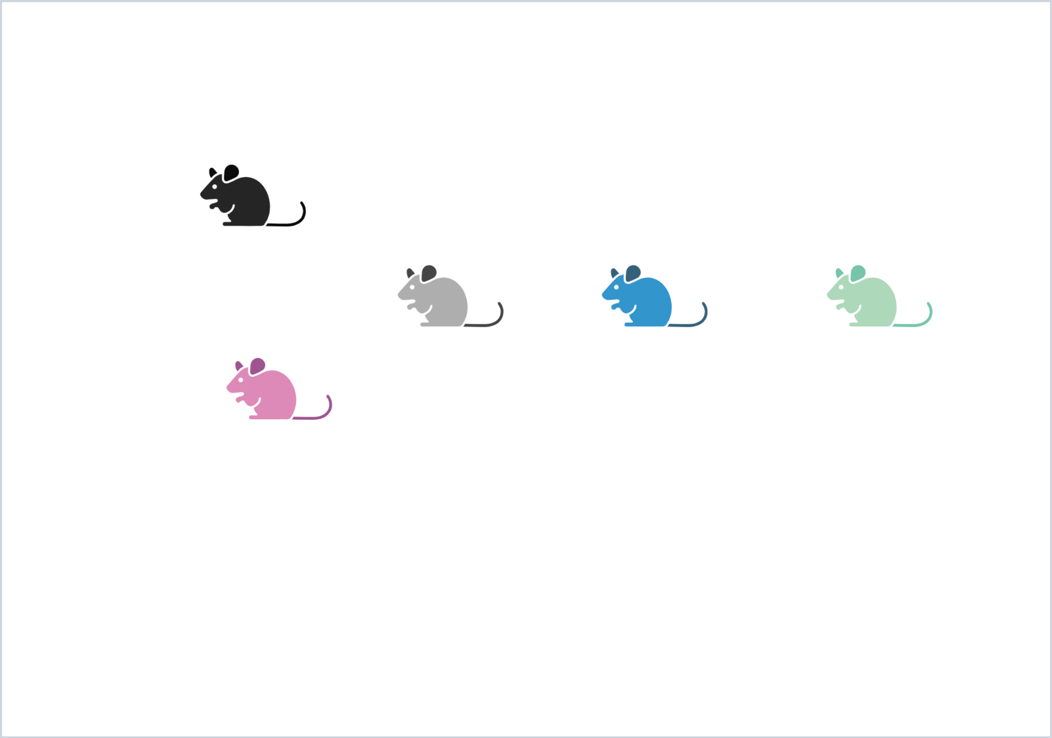

For any questions regarding this document, or other questions about publishing with BioRender, please refer to our BioRender Publication Guide, or contact BioRender Support at support@biorender.com.

49 Spadina Ave. Suite 200
Toronto ON M5V 2J1 Canada
www.biorender.com

Confirmation of Publication and Licensing Rights - Open Access

December 1st, 2025

Subscription Type: *Individual - Academic*
Agreement number: *UG292AVDKS*
Publisher Name: *JCI Insight*

Figure Title: *Fig.10E*

Citation to Use: *Created in BioRender. Qu, L. (2026) https://BioRender.com/rs679e9*

To whom this may concern,

This document ("Confirmation") hereby confirms that Science Suite Inc. dba BioRender ("BioRender") has granted the following BioRender user: Lintao Qu ("User") a BioRender Academic Publication License in accordance with BioRender's Terms of Service and Academic License Terms ("License Terms") to permit such User to do the following on the condition that all requirements in this Confirmation are met:

1) publish their Completed Graphics created in the BioRender Services containing both User Content and BioRender Content (as both are defined in the License Terms) in publications (journals, textbooks, websites, etc.); and
2) sublicense such Completed Graphics under "open access" publication sublicensing models such as CC-BY 4.0 and more restrictive models, so long as the conditions set forth herein are fully met.

Requirements of User:

1) All Completed Graphics to be published in any publication (journals, textbooks, websites, etc.) must be accompanied by the following citation either as a caption, footnote or reference for each figure that includes a Completed Graphic:
"Created in BioRender. Qu, L. (2026) https://BioRender.com/rs679e9 ".
2) All terms of the License Terms including all Prohibited Uses are fully complied with. E.g. For Academic License Users, no commercial uses (beyond publication in journals, textbooks or websites) are permitted without obtaining or switching to a BioRender Industry Plan.
3) A Reader (defined below) may request that the User allow their figure to be a public template for Readers to view, copy, and modify the figure. It is up to the User to determine what level of access to grant.

Open-Access Journal Readers:

Open-Access journal readers ("Reader") who wish to view and/or re-use a particular Completed Graphic in an Open-Access journal subject to CC-BY sublicensing may do so by clicking on the URL link in the applicable citation for the subject Completed Graphic.

The re-use/modification options below are available after the Reader requests the User to adapt their

figure as a BioRender template and the User has granted such access.

1) View-Only/Free Plan Use: A Reader who wishes to only view the Completed Graphic may do so in the BioRender Services as either a BioRender Free Plan user or simply as a viewer. By becoming a BioRender Free Plan user, the Reader may view, modify and re-use the Completed Graphic as permitted under BioRender's Basic License Terms (e.g. personal use only, no publishing or commercial use permitted).
2) Re-Use/Publish with No Modifications: For any re-use and re-publication of a Completed Graphic with no modification(s) to the Completed Graphic made by the Reader, a Reader may do so by citing the original author using the citation noted above with the Completed Graphic. The Reader must also comply with the underlying License Terms which apply to the Completed Graphic as noted above (e.g. no commercial use for Academic License).
3) Re-Use/Publish with Modifications: For any re-use and re-publication of a Completed Graphic with a modification(s) made by the Reader, the Reader may do so by becoming a BioRender user themselves under either an Academic or Industry Plan, citing the original author using the citation noted above with the Completed Graphic and complying with the applicable License Terms.

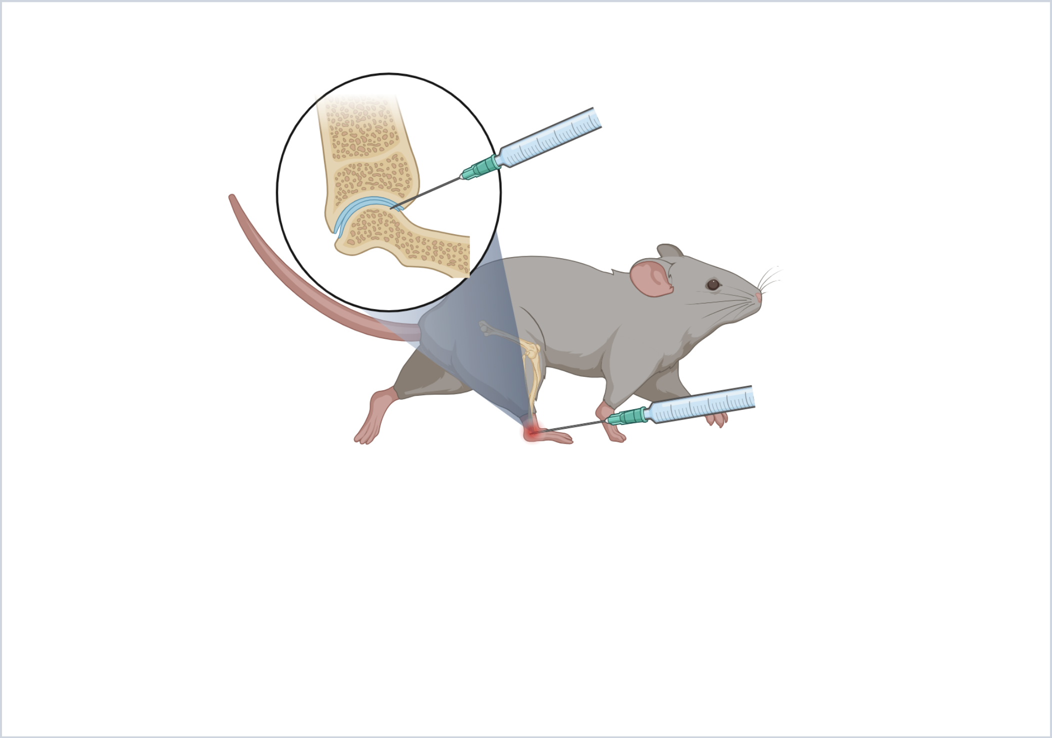

For any questions regarding this document, or other questions about publishing with BioRender, please refer to our BioRender Publication Guide, or contact BioRender Support at support@biorender.com.

49 Spadina Ave. Suite 200
Toronto ON M5V 2J1 Canada
www.biorender.com

Confirmation of Publication and Licensing Rights - Open Access

December 1st, 2025

Subscription Type:	*Individual - Academic*
Agreement number:	*QC292AVVQY*
Publisher Name:	*JCI Insight*
Figure Title:	*Graph Abstract*
Citation to Use:	*Created in BioRender. Qu, L. (2026) https://BioRender.com/jcyfh1q*

To whom this may concern,

This document ("Confirmation") hereby confirms that Science Suite Inc. dba BioRender ("BioRender") has granted the following BioRender user: Lintao Qu ("User") a BioRender Academic Publication License in accordance with BioRender's Terms of Service and Academic License Terms ("License Terms") to permit such User to do the following on the condition that all requirements in this Confirmation are met:

1) publish their Completed Graphics created in the BioRender Services containing both User Content and BioRender Content (as both are defined in the License Terms) in publications (journals, textbooks, websites, etc.); and
2) sublicense such Completed Graphics under "open access" publication sublicensing models such as CC-BY 4.0 and more restrictive models, so long as the conditions set forth herein are fully met.

Requirements of User:

1) All Completed Graphics to be published in any publication (journals, textbooks, websites, etc.) must be accompanied by the following citation either as a caption, footnote or reference for each figure that includes a Completed Graphic:
"Created in BioRender. Qu, L. (2026) https://BioRender.com/jcyfh1q ".
2) All terms of the License Terms including all Prohibited Uses are fully complied with. E.g. For Academic License Users, no commercial uses (beyond publication in journals, textbooks or websites) are permitted without obtaining or switching to a BioRender Industry Plan.
3) A Reader (defined below) may request that the User allow their figure to be a public template for Readers to view, copy, and modify the figure. It is up to the User to determine what level of access to grant.

Open-Access Journal Readers:

Open-Access journal readers ("Reader") who wish to view and/or re-use a particular Completed Graphic in an Open-Access journal subject to CC-BY sublicensing may do so by clicking on the URL link in the applicable citation for the subject Completed Graphic.

The re-use/modification options below are available after the Reader requests the User to adapt their

figure as a BioRender template and the User has granted such access.

1) <u>View-Only/Free Plan Use:</u> A Reader who wishes to only view the Completed Graphic may do so in the BioRender Services as either a BioRender Free Plan user or simply as a viewer. By becoming a BioRender Free Plan user, the Reader may view, modify and re-use the Completed Graphic as permitted under BioRender's [Basic License Terms](#) (e.g. personal use only, no publishing or commercial use permitted).
2) <u>Re-Use/Publish with No Modifications:</u> For any re-use and re-publication of a Completed Graphic with no modification(s) to the Completed Graphic made by the Reader, a Reader may do so by citing the original author using the citation noted above with the Completed Graphic. The Reader must also comply with the underlying License Terms which apply to the Completed Graphic as noted above (e.g. no commercial use for Academic License).
3) <u>Re-Use/Publish with Modifications:</u> For any re-use and re-publication of a Completed Graphic with a modification(s) made by the Reader, the Reader may do so by becoming a BioRender user themselves under either an Academic or Industry Plan, citing the original author using the citation noted above with the Completed Graphic and complying with the applicable License Terms.

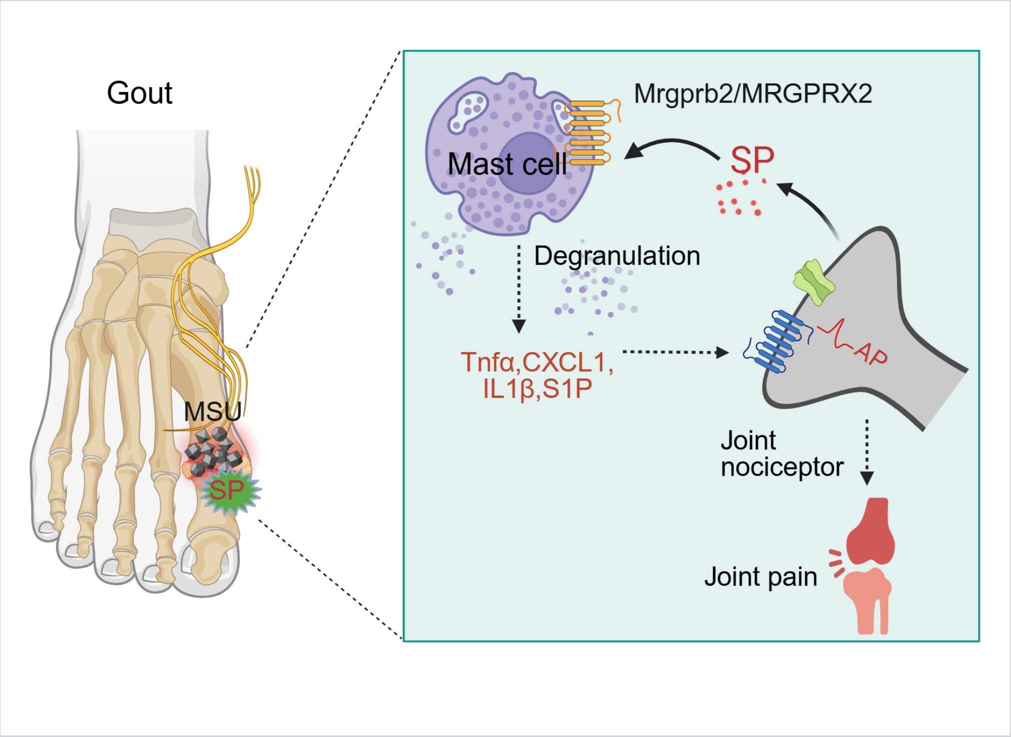

For any questions regarding this document, or other questions about publishing with BioRender, please refer to our [BioRender Publication Guide](#), or contact BioRender Support at [support@biorender.com](#).
